# On the Spectral Form Factor for Random Matrices

**DOI:** 10.1007/s00220-023-04692-y

**Published:** 2023-03-23

**Authors:** Giorgio Cipolloni, László Erdős, Dominik Schröder

**Affiliations:** 1 Princeton Center for Theoretical Science, Princeton University, Princeton, NJ 08544 USA; 2 IST Austria, Am Campus 1, 3400 Klosterneuburg, Austria; 3 Institute for Theoretical Studies, ETH Zurich, Clausiusstr. 47, 8092 Zurich, Switzerland

## Abstract

In the physics literature the spectral form factor (SFF), the squared Fourier transform of the empirical eigenvalue density, is the most common tool to test universality for disordered quantum systems, yet previous mathematical results have been restricted only to two exactly solvable models (Forrester in J Stat Phys 183:33, 2021. 10.1007/s10955-021-02767-5, Commun Math Phys 387:215–235, 2021. 10.1007/s00220-021-04193-w). We rigorously prove the physics prediction on SFF up to an intermediate time scale for a large class of random matrices using a robust method, the multi-resolvent local laws. Beyond Wigner matrices we also consider the monoparametric ensemble and prove that universality of SFF can already be triggered by a single random parameter, supplementing the recently proven Wigner–Dyson universality (Cipolloni et al. in Probab Theory Relat Fields, 2021. 10.1007/s00440-022-01156-7) to larger spectral scales. Remarkably, extensive numerics indicates that our formulas correctly predict the SFF in the entire *slope-dip-ramp* regime, as customarily called in physics.

## Introduction

Spectral statistics of disordered quantum systems tend to exhibit universal behavior and hence are widely used to study quantum chaos and to identify universality classes. In the chaotic regime, the celebrated Wigner–Dyson–Mehta eigenvalue gap statistics involving the well-known *sine-kernel* [42] tests this universality on the scale of individual eigenvalue spacing. On this small *microscopic* scale the universality phenomenon is the most robust and it depends only on the fundamental symmetry type of the model. On larger scales more details of the model influence the spectral statistics, nevertheless several qualitative and also quantitative universal patterns still prevail.

### The spectral form factor and predictions from physics

In the physics literature the standard tool to investigate eigenvalues $\lambda _1, \lambda _2, \ldots , \lambda _N$ of a Hermitian $N\times N$ matrix (Hamiltonian) *H* on all scales at once is the *spectral form factor* (SFF) [40] defined as

1.1 $$\begin{aligned} {{\,\mathrm{\textbf{SFF}}\,}}(t): = \frac{1}{N^2} \sum _{i,j=1}^N e^{\textrm{i}t(\lambda _i-\lambda _j)}=|{\langle {e^{\textrm{i}t H}} \rangle } |^2 \end{aligned}$$

with a real time parameter $t>0$, i.e. it is the square of the Fourier transform of the empirical spectral density. Here we denoted the normalized trace of any $N\times N$ matrix *A* by $\langle {A} \rangle = \frac{1}{N}{{\,\textrm{Tr}\,}}A$. In case of random *H*, the expectation of ${{\,\mathrm{\textbf{SFF}}\,}}(t)$ is denoted by

1.2 $$\begin{aligned} K(t):={\textbf{E}}\big [ {{\,\mathrm{\textbf{SFF}}\,}}(t)\big ]. \end{aligned}$$

For typical disordered Hamiltonians a key feature of ${{\,\mathrm{\textbf{SFF}}\,}}(t)$ is that for larger *t* (more precisely, in the *ramp* and *plateau* regimes, see later) it is strongly dependent on the sample, i.e. the standard deviation of ${{\,\mathrm{\textbf{SFF}}\,}}(t)$ is comparable with *K*(*t*). In other words, ${{\,\mathrm{\textbf{SFF}}\,}}(t)$ is *not* self-averaging [45] despite the large summation in (1.1).

The spectral form factor and its expectation *K*(*t*) have a very rich physics literature since they contain most physically relevant information about spectral statistics. Quantizations of integrable systems typically result in $K(t)\sim 1/N$ for all *t* where *N* is the dimension of the Hilbert space. Chaotic systems give rise to a linearly growing behavior of *K*(*t*) for smaller *t* (so-called *ramp*) until it turns into a flat regime, the *plateau*. The turning point is around the Heisenberg time $T_H$, but the details of the transition depend on the symmetry class of *H* and on whether the eigenvalues are rescaled to take into account the non-constant density of states (in physics terminology: *unfolding the spectrum*). For example, in the time irreversible case (GUE symmetry class) the unfolded SFF has a sharp kink, while in the GOE symmetry class the kink is smoothened. The exact formulas can be computed from the Fourier transform of the two point eigenvalue correlation function of the corresponding Gaussian random matrix ensemble, see [42, Eqs. (6.2.17), (7.2.46)], the result is

1.3 $$\begin{aligned} K_\textrm{GUE}(\tau T_H) \approx \frac{1}{N}\times {\left\{ \begin{array}{ll} \tau , &{} 0< \tau \le 1 \\ 1, &{} \tau \ge 1 \end{array}\right. }, \qquad K_{\textrm{GOE}}(\tau T_H) \approx \frac{1}{N}\times {\left\{ \begin{array}{ll} 2\tau - \tau \log (1+2\tau ), &{} 0< \tau \le 1 \\ 2- \tau \log \frac{2\tau +1}{2\tau -1},&{} \tau \ge 1 \end{array}\right. }, \nonumber \\ \end{aligned}$$

for any fixed $\tau >0$ in the large *N* limit. Here we expressed the physical time *t* in units of the Heisenberg time, $\tau = t/T_H$, where $T_H$ is given by $T_H = 2\pi {\bar{\rho }}$ with ${\bar{\rho }}$ being the average density. Choosing the standard normalisation for the independent (up to symmetry) matrix elements,

1.4 $$\begin{aligned} {\textbf{E}}h_{ij}=0, \qquad {\textbf{E}}|h_{ij}|^2 =\frac{1}{N}, \end{aligned}$$

the limiting density of states is the semicircle law $\rho _{\textrm{sc}}(E)=\frac{1}{2\pi }\sqrt{(4-E^2)_+}$, so we have *N* eigenvalues in an interval of size 4, hence ${\bar{\rho }} = N/4$ and thus $T_H= \frac{\pi }{2}N$. In particular, in the original *t* variable

1.5 $$\begin{aligned} K_\textrm{GUE}(t) \approx {\left\{ \begin{array}{ll} \frac{2t}{\pi N^2}, &{} \delta N\le t\le \frac{\pi }{2}N \\ \frac{1}{N}, &{} t\ge \frac{\pi }{2}N. \end{array}\right. } \end{aligned}$$

Note the lower bound on *t*: the formula holds in the large *N* limit in the regime where $t\ge \delta N$ for some fixed $\delta >0$ that is independent of *N*. The corresponding formulas without unfolding the spectrum (i.e. for the quantity defined in (1.1)) are somewhat different, see e.g. [9, Eq. (4.8)] for the GUE case; they still have a ramp-plateau shape but the kink is smoothened.

The ramp-plateau picture and its sensitivity to the symmetry type has been established well beyond the standard mean field random matrix models. In fact, the Bohigas-Giannoni-Schmit conjecture [6] asserts that the formulas (1.3) are universal, i.e. they hold essentially for any chaotic quantum system, depending only on whether the system is without or with time reversal symmetry. The nonrigorous but remarkably effective semiclassical orbit theory [4, 31, 43, 48] based upon Gutzwiller’s trace formula [27] and many follow-up works verified this conjecture for quantizations of a large family of classical chaotic systems, e.g. for certain billiards.

**Fig. 1:**
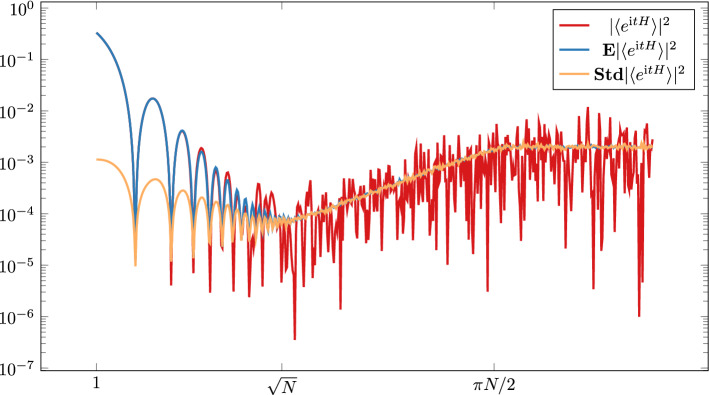
A typical *slope-dip-ramp-plateau* picture for the spectral form factor of a chaotic system. The figure on log-log scale shows the SFF of a single GUE realisation $H$ of size $500\times 500$, as well as the empirical mean and standard deviation obtained from $500$ independent realisations

For smaller times, $t\ll T_H$, other details of *H* may become relevant. In particular the drop from $K(t=0)=1$ to $K(t)\ll 1$ for $1\ll t\ll T_H$ is first dominated by the typical non-analyticity of the density of states at the spectral edges giving rise to the *slope regime* up to an intermediate minimum point of *K*(*t*), called the *dip* (in the early literature the dip was called *correlation hole* [40], for a recent overview, see [17]).

Figure 1 shows the typical *slope-dip-ramp-plateau* picture for the GUE ensemble. Formula (1.5) is valid starting from scales $t\gg N^{1/2}$, while *K*(*t*) is oscillatorily decreasing for $t\lesssim N^{1/2}$ with a dip-time $t_{\textrm{dip}}\sim N^{1/2}$. Thus *K*(*t*) follows the universal behavior (1.5) only for $t\gg t_{\textrm{dip}}$. In this regime the fluctuation of the SFF is comparable with its expectation, *K*(*t*), in fact $\langle {e^{itH}} \rangle$ is approximately Gaussian. In contrast, the dominant contribution to the slope regime, $t\ll t_{\textrm{dip}}$, is self-averaging with a relatively negligible fluctuation. However, if the edge effects are properly discounted (e.g. by considering the circular ensemble with uniform spectral density on the unit circle), i.e. the slope regime is entirely removed, then the Gaussian behavior holds for all $t\ll T_H$ with a universal variance given by (1.5).

In more recent works spectral form factors were studied for the celebrated Sachdev-Ye-Kitaev (SYK) model [18, 23, 24, 32, 46] which also exhibits a similar *slope-dip-ramp-plateau* pattern although the details are still debated in the physics literature and the numerics are much less reliable due to the exponentially large dimensionality of the model.

### Our results

Quite surprisingly, despite its central role in the physics literature on quantum chaos, SFF has not been rigorously investigated in the mathematics literature up to very recently, when Forrester computed the large *N* limit of *K*(*t*) rigorously for the GUE in [21] and the Laguerre Unitary Ensemble (LUE) in [22] in the entire regime $t\ll N$. Both results rely on a remarkable identity from [9] (and its extension to the LUE case) and on previous stimulating work of Okuyama [44]. However, these methods use exact identities and thus are restricted to a few explicitly solvable invariant ensembles.

The main goal of the current paper is to investigate SFF beyond these special cases with a robust method, the multi-resolvent local laws. While our approach is valid for quite general ensembles, for definiteness we focus on two models: the standard Wigner ensemble (for both symmetry classes) and the novel *monoparametric ensemble* introduced recently [25] by Gharibyan, Pattison, Shenker and Wells. The latter consists of matrices of the form $H^s:= s_1 H_1+s_2H_2$, where $H_1$ and $H_2$ are typical but *fixed* realisations of two independent Wigner matrices and $s=(s_1, s_2)\in S^1\subset {\textbf{R}}$ is a continuous random variable. The normalization $s_1^2+s_2^2=1$ guarantees that the semicircle law for $H^s$ is independent of *s* and it also shows that the model has effectively only one random parameter. One may also consider similar ensembles with finitely many parameters (see Remark 2.4) resulting in qualitatively the same behavior but with different power laws, see Table 1.

We study the statistics of $H^s$ in the probability space of the single random variable *s* and probe how much universality still persists with such reduced randomness. We write ${\textbf{E}}_s$ for the expectation wrt. *s* and ${\textbf{E}}_H$, ${{\,\mathrm{\textbf{Std}}\,}}_H$ for the expectation and standard deviation wrt. $H_1$ and $H_2$.

Our main result is to prove a formula for the expectation and standard deviation of SFF for both ensembles up to an intermediate time. While this does not include the ramp regime, it already allows us to draw the following two main conclusions of the paper:

- The expectation and standard deviation of ${{\,\mathrm{\textbf{SFF}}\,}}(t)$ for Wigner and monoparametric ensembles exhibit the same universal behavior to leading order for $1\ll t\ll N^{1/4}$ if the trivial edge effects are removed. In the monoparametric case it is quite remarkable that already a single real random variable generates universality.
- For the monoparametric ensemble $K(t)={\textbf{E}}_s [{{\,\mathrm{\textbf{SFF}}\,}}(t)]$ depends non-trivially on the fixed $H_1, H_2$ matrices, but for large *t* this dependence is a subleading effect whose relative size becomes increasingly negligible as a negative power of *t*. In particular, while the speed of convergence to universality is much slower for the monoparametric ensemble than for the Wigner case, it is improving for larger *t*.

The second item answers a question raised by the authors of [25] which strongly motivated the current work. In particular, sampling from *s* does not give a consistent estimator for *K*(*t*), but the relative precision of such estimate improves for larger times.

We supplement these proofs with an extensive numerics demonstrating that both conclusions hold not only for $t\ll N^{1/4}$ but for the entire ramp regime, i.e. up to $t\ll T_H\sim N$. Note that recently we have proved [15] that the Wigner–Dyson–Mehta eigenvalue gap universality holds for the monoparametric ensemble, which strongly supports, albeit does not prove, that *K*(*t*) in the plateau regime is also universal.

We remark that our method applies without difficulty for finite temperatures (expressed by a parameter $\beta >0$) and for different-time autocorrelation functions, i.e. for

$$\begin{aligned} \langle {e^{(-\beta +\textrm{i}t) H}} \rangle \langle { e^{(-\beta -\textrm{i}t')H}} \rangle \end{aligned}$$

as well, but for the simplicity of the presentation we focus on ${{\,\mathrm{\textbf{SFF}}\,}}(t)$ defined in (1.1), i.e. on $\beta =0$ and $t=t'$.

### Relations to previous mathematical results

Rigorous mathematics for the spectral form factor, even for Wigner matrices or even for GOE, significantly lags behind establishing the compelling physics picture about the slope-dip-ramp-plateau. Given the recently developed tools in random matrix theory, it may appear surprising that they do not directly answer the important questions on SFF. We now briefly explain why.

#### Limitations of the resolvent methods

For problems on macroscopic spectral scales (involving the cumulative effect of order *N* many eigenvalues), and to a large extent also on mesoscopic scales (involving many more than *O*(1) eigenvalues), the *resolvent method* is suitable. This method considers the resolvent $G(z)= (H-z)^{-1}$ of *H* for a spectral parameter *z* away from (but typically still close to) the real axis and establishes that in a certain sense *G*(*z*) becomes deterministic. This works for $\eta =\Im z\gg N^{-1}$ (in the bulk spectrum), i.e. on scales just above the eigenvalue spacing (note that the imaginary part of the spectral parameter sets a scale in the spectrum). Such results are called *local laws* and they can be extended to regular functions *f*(*H*) by standard spectral calculus (Helffer–Sjöstrand formula, see (3.3) later).

However, the interesting questions about SFF concern a 1/*N* subleading fluctuation effect beyond the local laws. Indeed

$$\begin{aligned} {{\,\textrm{Tr}\,}}e^{\textrm{i}tH} = \sum _i e^{\textrm{i}t \lambda _i} \end{aligned}$$

is a special case of the well-studied *linear eigenvalue statistics*, ${{\,\textrm{Tr}\,}}f(H)=\sum _i f(\lambda _i)$, with the regular test function $f(\lambda )= e^{\textrm{i}t\lambda }$. To leading order it is deterministic and its fluctuation satisfies the central limit theorem (CLT) without the customary $\sqrt{N}$ normalisation, i.e.

1.6 $$\begin{aligned} \sum _i f(\lambda _i) - {\textbf{E}}\sum _i f(\lambda _i)\approx {\mathcal {N}}\big (0, V_f), \qquad \text{ with }\quad {\textbf{E}}\sum _i f(\lambda _i) = N \int _{\textbf{R}}f(x) \rho _\textrm{sc}(x){\text {d}}\!{}x + O_f(1). \end{aligned}$$

is a normal random variable with variance.^1^

1.7 $$\begin{aligned} V_f = \frac{1}{4\pi ^2}\iint _{-2}^2 |{\frac{f(x)-f(y)}{x-y}} |^2\frac{4-xy}{\sqrt{4-x^2}\sqrt{4-y^2}}{\text {d}}\!{}x {\text {d}}\!{}y. \end{aligned}$$

The computation of higher moments of ${{\,\textrm{Tr}\,}}f(H) - {\textbf{E}}{{\,\textrm{Tr}\,}}f(H)$ requires a generalization of the local laws to polynomial combinations of several *G*’s that are called *multi-resolvent local laws*.

Applying (1.6)–(1.7) to $f(x)= e^{\textrm{i}tx}$ we obtain, roughly,

1.8 $$\begin{aligned} {{\,\mathrm{\textbf{SFF}}\,}}(t) = \frac{1}{N^2}\big | {{\,\textrm{Tr}\,}}e^{\textrm{i}t H}\big |^2 \approx \Big [\frac{J_1(2t)}{t} + O\Big ( \sqrt{\frac{V_f}{N^2} } \Big ) \Big ]^2, \qquad t\gg 1, \end{aligned}$$

using that

$$\begin{aligned} \int _{\textbf{R}}f(x) \rho _\textrm{sc}(x){\text {d}}\!{}x = \int _{\textbf{R}}e^{\textrm{i}t x} \rho _\textrm{sc}(x){\text {d}}\!{}x = \frac{J_1(2t)}{t}, \end{aligned}$$

where $J_1$ is the first Bessel function of the first kind. Note that $V_f$ in (1.7) scales essentially as the $H^{1/2}$ Sobolev norm of *f* hence $V_f\sim t$ for our $f(x)= e^{\textrm{i}tx}$ in the regime $t\gg 1$. Therefore the size of the fluctuation term in (1.8) is $V_f/N^2\sim t/N^2$ and it competes with the deterministic term $(J_1/t)^2\sim t^{-3}$. The dip time $t_{\textrm{dip}}\sim \sqrt{N}$ is obtained as the threshold where the fluctuation (the linear ramp function) becomes bigger than the slope function $(J_1/t)^2$. This argument, however, is heuristic as it neglects the error terms in (1.6) that also depend on *t* via *f*.

CLT for linear statistics (1.6) for Wigner matrices *H* has been proven [1, 3, 13, 26, 28–30, 33, 34, 36, 38, 41, 47, 49] for test functions of the form $$ f(x)= g(N^a(x-E))$$ with some fixed reference point $$|E|<2$$, scaling exponent $$a\in [0, 1)$$ and smooth function *g* with compact support, i.e for macroscopic ($$a=0$$) and mesoscopic ($$0<a<1$$) test functions living on a *single* scale $$N^{-a}$$. These proofs give optimal error terms for such functions but they were not optimized for dealing with functions that oscillate on a mesoscopic scale *and* have macroscopic support, like $$f(x)= e^{\textrm{i}t x}$$ for some $$t\sim N^\alpha $$, $$\alpha >0$$. The only CLT-type result for a special two-scale observable is in [2] where the eigenvalue counting function smoothed on an intermediate scale $$N^{-1/3}$$ was considered.

Quite remarkably, extensive numerics shows that the formulas (1.6)–(1.7) for $f(x)= e^{\textrm{i}t x}$ are in perfect agreement with the expected behavior of *K*(*t*) in the entire slope-dip-ramp regime all the way up to $t\ll N$, i.e. the CLT for linear statistics correctly predicts SFF well beyond its proven regime of validity. In the current paper we optimise the error terms specifically for $e^{\textrm{i}tx}$ and thus we could cover the regime $t\ll N^{5/11}$ for the variance in (1.6) (corresponding to ${\textbf{E}}[ {{\,\mathrm{\textbf{SFF}}\,}}(t)]$).

#### Limitations of Dyson Brownian motion techniques

For the microscopic scale (i.e. comparable with the eigenvalue spacing, 1/*N* in the bulk) the resolvent is heavily fluctuating as it strongly depends on single eigenvalues. Local laws cannot access them, but in this regime another approach, the careful analysis of the *Dyson Brownian Motion (DBM)* becomes applicable. While these two approaches are complementary and apparently cover all scales, the actual methods require additional conditions that seriously restrict their use for SFF.

The formulas (1.3) are obtained by computing the Fourier transform of the two point correlation function of the rescaled *(unfolded)* eigenvalues. Indeed, in the GUE case $K_\textrm{GUE}(t)$ in (1.3) is just the Fourier transform of $p_2(x,y) - 1+\delta (x-y)$ in the difference variable $x-y$, where

$$\begin{aligned} p_2(x,y):= 1- \Big (\frac{\sin (\pi (x-y))}{\pi (x-y)} \Big )^2, \end{aligned}$$

is the two point function, given by the celebrated Wigner–Dyson sine kernel, and $K_{\textrm{GOE}}(t)$ has a similar origin. Wigner–Dyson theory is designed for microscopic scales, i.e. to describe eigenvalue correlations on scales comparable with the local level spacing $\Delta$, this is encoded in the fact that (1.3) holds for any fixed $\tau >0$ in the $N\rightarrow \infty$ limit (equivalently that (1.5) holds only for $t\ge \delta N$ since $\Delta \sim 1/N$ in the bulk). While this is a very elegant argument supporting (1.3), mathematically it is quite far from a rigorous proof.

The mathematical proofs of the sine-kernel universality use test functions that are rapidly decaying beyond scale $\Delta$. The typical statements (so called *fixed energy universality* [7, 39]) show that for any fixed energy *E* in the bulk

$$\begin{aligned} \sum _{i<j} g\big ( N\rho _\textrm{sc}(E)(\lambda _i-E), N\rho _\textrm{sc} (E)(\lambda _j-E) \big ) \rightarrow \iint _{\textbf{R}}g(x,y) p_2(x,y){\text {d}}\!{}x{\text {d}}\!{}y \end{aligned}$$

in the large *N* limit, for any smooth, compactly supported functions $g:{\textbf{R}}^2\rightarrow {\textbf{R}}$. The current methods for proving the Wigner–Dyson universality cannot deal with functions that are macroscopically supported, like $g(x,y)= e^{\textrm{i}t(x-y)}$ with a fast oscillation $t\sim N$.

### Summary

Using multi-resolvent local laws we prove a CLT for linear statistics of monoparametric ensembles (Theorem 2.5) with covariance

$$\begin{aligned} {{\,\mathrm{\textbf{Cov}}\,}}( {{\,\textrm{Tr}\,}}f(H^s), {{\,\textrm{Tr}\,}}g(H^r) ) \approx \frac{1}{\pi ^2}\iint f'(x) g'(y) \log |{\frac{ 1-\langle {s,r} \rangle m_\textrm{sc}(x)\overline{m_\textrm{sc}(y)}}{1- \langle {s,r} \rangle m_\textrm{sc}(x)m_\textrm{sc}(y)}} | {\text {d}}\!{}x {\text {d}}\!{}y \end{aligned}$$

with an additional term depending on the fourth cumulant. Due to a careful analysis of the error terms this allows us to prove the expected behavior on the expectation and standard deviation of the SFF for Wigner matrices for $t\ll N^{5/17}$ (Theorem 2.7) and for the monoparametric ensemble for $t\ll N^{1/4}$ (Theorem 2.8). Beyond these regime the spectral form factor is not understood mathematically apart from the special GUE and LUE cases. However, we can still use our predictions from the CLT for linear statistics (1.6) to derive an Ansatz for the behavior of ${{\,\mathrm{\textbf{SFF}}\,}}(t)$ in the entire $t\ll N$ regime. In particular, we show that the SFF is universal for the monoparametric ensemble. We find numerically that our theory correctly reproduces ${{\,\mathrm{\textbf{SFF}}\,}}(t)$ for any $t\ll N$ and it also coincides with the physics predictions for the GUE case.

### Notations and conventions

For positive quantities *f* and *g* we will frequently use the notation $f\approx g$ meaning that $f/g \rightarrow 1$ in a limit that is always clear from the context. Similarly, $f\ll g$ means that $f/g\rightarrow 0$. Finally, the relation $f\sim g$ means that there exist two positive constants *c*, *C* such that $c\le f/g \le C$.

We say that an event holds “with very high probability” if for any fixed $D>0$ the probability of the event is bigger than $1-N^{-D}$ if $N\ge N_0(D)$, for some $N_0(D)>0$.

## Statement of the Main Results

Our new results mainly concern the monoparametric ensemble but for comparison reasons we also prove the analogous results for the Wigner ensemble. We start with the two corresponding definitions.

### Definition 2.1

The Wigner ensemble consists of Hermitian $N\times N$ random matrices *H* with the following properties. The off-diagonal matrix elements below the diagonal are independent, identically distributed (i.i.d) real ($\beta =1$) or complex $(\beta =2)$ random variables; in the latter case we assume that ${\textbf{E}}h_{ij}^2=0$. The diagonal elements are i.i.d. real random variables with ${\textbf{E}}h_{ii}^2 = 2/(N\beta )$. Besides the standard normalisation (1.4), we also make the customary moment assumption: for every $q\in {\textbf{N}}$ there is a constant $C_q$ such that

1 $$\begin{aligned} {\textbf{E}}\big | \sqrt{N} h_{ij}\big |^{q}\le C_q. \end{aligned}$$

In the case of Gaussian distributions, it is called the Gaussian Orthogonal or Unitary Ensemble (GOE/GUE), for the real and complex cases, respectively.

### Remark 2.2

The assumptions ${\textbf{E}}h_{ij}^2=0$ in the complex case, and ${\textbf{E}}h_{ii}^2=2/(\beta N)$ are made purely for convenience. All results can easily be generalised beyond this case but we refrain from doing so for notational simplicity.

### Definition 2.3

The monoparametric ensemble consists of Hermitian $N\times N$ random matrices of the form

2.1 $$\begin{aligned} H= H^s:=s_1H_1+s_2H_2, \end{aligned}$$

where $H_1, H_2$ are independent Wigner matrices satisfying^2^${\textbf{E}}|{ h_{ij}^{(1)}} |^4={\textbf{E}}|{ h_{ij}^{(2)}} |^4$ and $s=(s_1,s_2)\in S^1$ is a random vector, independent of $H_1, H_2$. On the distribution of *s* we assume that it has an square integrable density $\rho (s)$ independent of $N$. We write ${\textbf{E}}_s$ for the expectation wrt. *s* and ${\textbf{E}}_H$, ${{\,\mathrm{\textbf{Std}}\,}}_H$ for the expectation and standard deviation wrt. the Wigner matrices $H_1$ and $H_2$.

The parameter space $S^1\subset {\textbf{R}}^2$ inherits the usual scalar product and norms from ${\textbf{R}}^2$, so for $s,r\in S^1$ we have

$$\begin{aligned} \langle {s,r} \rangle :=s_1r_1+s_2r_2,\quad \Vert s\Vert _p:=(|{s_1} |^p+|{s_2} |^p)^{1/p}. \end{aligned}$$

We also introduce the entrywise product of two vectors:

$$\begin{aligned} s\odot r:=(s_1r_1,s_2r_2). \end{aligned}$$

For a fixed *s*, $H^s$ is just the weighted sum of two Wigner matrices, and, due to the normalisation, itself is just a Wigner matrix. However, the concept of monoparametric ensemble views $H^s$ as a random matrix in the probability space of the single random variable *s* for a typical but fixed (quenched) realization of $H_1$ and $H_2$. While Wigner matrices have a large $(\sim N^2)$ number of independent random degrees of freedom, the monoparametric ensemble is generated by one single random variable hence, naively, much less universality properties are expected. Nevertheless, the standard Wigner–Dyson local eigenvalue universality holds [15].

### Remark 2.4

In [15] we considered the un-normalized monoparametric model $H^s:=H_1+sH_2$, for some real valued random variable *s*, whose density of states is a rescaled semicircular distribution. In this paper we prefer to work with more homogeneous models since the formulas are somewhat nicer, but our main results also apply to this inhomogeous model with some slightly different exponents in the error terms. One may also consider a different un-normalized ensemble, $s_1H_1+s_2H_2$ with $s\in {\textbf{R}}^2$ having an absolutely continuous distribution, which is effectively a two parameter model.

Similar results also hold for the multi-parametric analogue of (22.2), i.e. $s_1H_1+\cdots +s_kH_k$ for $s\in S^{k-1}$, see Remark 2.6 and Sect. 2.4 later. Despite all these options, for definiteness, the main body of this paper concerns the homogenous monoparametric model from Definition 2.3.

### Central limit theorem for sum of Wigner matrices

To understand the effect of the random *s*, we study the joint statistics of $H^s$ and $H^r$ for two different fixed realisations *r*, *s* in the probability space of $H_1, H_2$, i.e. we aim at the correlation effects between $H^s$ and $H^r$. We introduce the short-hand notations

2 $$\begin{aligned} \langle {f} \rangle _\textrm{sc}:= & {} \int _{-2}^2 f(x)\frac{\sqrt{4-x^2}}{2\pi }{\text {d}}\!{}x, \quad \langle {f} \rangle _\mathrm {1/sc}:=\int _{-2}^2 f(x)\frac{1}{\pi \sqrt{4-x^2}}{\text {d}}\!{}x, \nonumber \\{} & {} \kappa _4:= N^2 {\textbf{E}}|{h_{12}} |^4 -1-\frac{2}{\beta }. \end{aligned}$$

To estimate the error term in the following theorem we introduce a parameter $1\le \tau \ll N$ and the weighted norm

2.2 $$\begin{aligned} \Vert f\Vert _\tau :=\tau ^2\Vert f\Vert _\infty +\tau \Vert f\Vert _{H^1}+\Vert f\Vert _{H^2}, \end{aligned}$$

where $\Vert f\Vert _{H^k}^2:=\sum _{j\le k}\int _{\textbf{R}}|{f^{(j)}} |^2$ is the usual Sobolev norm. For the applications later, the parameter $\tau$ will be optimized.

#### Theorem 2.5

For $s\in S^1$ and test functions $f\in H^2({\textbf{R}})$ the family of random variables ${{\,\textrm{Tr}\,}}f(H^s)$ is approximately Gaussian of mean

2.3 $$\begin{aligned} {\textbf{E}}{{\,\textrm{Tr}\,}}f(H^s)= & {} N\langle {f} \rangle _\textrm{sc} + \kappa _4\Vert s\Vert _4^4 \langle {\frac{x^4-4x^2+2}{2}f} \rangle _\mathrm {1/sc} \nonumber \\{} & {} +\, \varvec{1}(\beta =1)\Bigl [\frac{f(2)+f(-2)}{4} -\frac{\langle {f} \rangle _\mathrm {1/sc}}{2}\Bigr ] + {{\mathcal {O}}}({{\mathcal {E}}_1}), \end{aligned}$$

and fluctuation

2.4 $$\begin{aligned} {\textbf{E}}\prod _{i=1}^p \Bigl ({{\,\textrm{Tr}\,}}f_i(H^{s^i})-{\textbf{E}}{{\,\textrm{Tr}\,}}f_i(H^{s^i})\Bigr ) = \sum _{P\in \textrm{Pair}([p])} \prod _{(i,j)\in P} v^{s^is^j}(f_i,f_j) + {\mathcal {O}}_p({\mathcal {E}}_p), \end{aligned}$$

for any fixed $p\in {\textbf{N}}$, functions $f_1,\dots , f_p\in H^2({\textbf{R}})$, and parameters $s^1,\dots , s^p\in S^1$, where^3^

5 $$\begin{aligned} v^{sr}(f,g)&:= \frac{1}{\beta \pi ^2} \iint _{-2}^2 f'(x)g'(y) V^{sr}(x,y){\text {d}}\!{}x {\text {d}}\!{}y + \frac{\kappa _4}{2} \langle {s\odot s,r \odot r} \rangle \langle {(2-x^2)f} \rangle _\mathrm {1/sc}^2 \nonumber \\ V^{sr}(x,y)&:= \log |{1-\langle {s,r} \rangle m_\textrm{sc}(x)\overline{m_\textrm{sc}(y})} | - \log |{1-\langle {s,r} \rangle m_\textrm{sc}(x)m_\textrm{sc}(y)} |. \end{aligned}$$

Here ${\mathcal {E}}_p$ are error terms which for any $1\le \tau \ll N$ and any $\xi ,\epsilon >0$ may be estimated by^4^

2.5 $$\begin{aligned} {\mathcal {E}}_1:=\frac{N^\xi \Vert f\Vert _\tau }{N^{1/2}\tau ^{1/2}}, \quad {\mathcal {E}}_p:=N^\xi \left( \frac{1}{N^{1/2}\tau ^{3/2}}+\frac{N^\epsilon }{N} +\frac{N^{-\epsilon }}{\tau ^{2p-1}}\right) \left( 1+\frac{\tau ^2}{N^{1-2\epsilon }}\right) \prod _{i\in [p]}\Vert f_i\Vert _\tau , \end{aligned}$$

for $p\ge 2$. Additionally, if $s^1=\dots =s^p$, i.e. in the Wigner case, we have the improved bound

6 $$\begin{aligned} {\mathcal {E}}_p:= \frac{1}{N^{1/2}\tau ^{3/2}}\prod _{i\in [p]}\Vert f_i\Vert _\tau \end{aligned}$$

and the first term of (2.7) for $\beta =2$ coincides with (1.7).

We note that (2.7) generalizes the standard variance calculation yielding (1.7) to $s\ne r$, see Sect. 3.2.4.

#### Remark 2.6

Theorem 2.5 verbatim holds true also for the multi-parametric model

$$\begin{aligned}s_1H_1+\cdots +s_kH_k\end{aligned}$$

upon interpreting $\langle {s,r} \rangle$ and $\Vert s\Vert _p$ as the Euclidean inner product and $p$-norm in ${\textbf{R}}^k$. Similarly, Theorem 2.5 also applies to the un-normalised case $s\in {\textbf{R}}^2$ for which on the rhs. of (52.5) the function $f$ has to be replaced by $f(\Vert s\Vert \cdot )$ with $\Vert \cdot \Vert :=\Vert \cdot \Vert _2$ and $v^{sr}$ from (2.7) has to be replaced by

2.6 $$\begin{aligned} \begin{aligned} \widetilde{v}^{sr}(f,g)&:= \frac{\Vert s\Vert \Vert r\Vert }{\beta \pi ^2} \iint _{-2}^2 f'(\Vert s\Vert x)g'(\Vert r\Vert y) V^{\frac{s}{\Vert s\Vert },\frac{r}{\Vert r\Vert }}(x,y){\text {d}}\!{}x {\text {d}}\!{}y \\&\qquad + \frac{\kappa _4}{2} \langle {s\odot s,r \odot r} \rangle \langle {(2-x^2)f(\Vert s\Vert x)} \rangle _\mathrm {1/sc}\langle {(2-x^2)f(\Vert r\Vert x)} \rangle _\mathrm {1/sc}. \end{aligned} \end{aligned}$$

### SFF for Wigner and monoparametric ensemble

In this section we specialise Theorem 2.5 to the SFF case. We define the approximate expectation (rescaled by 1/*N*)

2.7 $$\begin{aligned} \begin{aligned} e_N^s(t)&:= e(t) + \frac{1}{N}\Bigl [\kappa _4\Vert s\Vert _4^4\left( 1-\frac{6}{t^2}\right) J_0(2 t)+\kappa _4\Vert s\Vert _4^4\left( \frac{6}{t^{3}}-\frac{4}{t}\right) J_1(2 t) - \varvec{1}(\beta =1)\frac{J_0(2 t) - \cos (2 t)}{2}\Bigr ] \\ e(t)&:=\frac{J_1(2t)}{t} \end{aligned} \nonumber \\ \end{aligned}$$

in terms of the Bessel functions $J_k$ of the first kind. We also define the approximate variance

8 $$\begin{aligned} \begin{aligned} v_{\pm ,\kappa }^{sr}(t)&:=v^{sr}(e^{\textrm{i}t \cdot },e^{\pm \textrm{i}t \cdot })=v_\pm ^{sr}(t)+\kappa _4 \langle {s\odot s,r\odot r} \rangle J_2(2t)^2,\\ v_\pm ^{sr}(t)&:= \frac{t^2}{\beta \pi ^2} \iint _{-2}^2 \cos \Bigl (t (x\pm y)\Bigr )V^{sr}(x,y){\text {d}}\!{}x {\text {d}}\!{}y, \end{aligned} \end{aligned}$$

From Theorem 2.5, choosing $f_i(x)=e^{\pm \textrm{i}tx}$ and $\tau =t$, and recalling that $\langle {e^{\pm \textrm{i}t H^s}} \rangle =N^{-1}\textrm{Tr}\, e^{\pm \textrm{i}t H^s}$, we readily conclude the following asymptotics for SFF of the Wigner and monoparametric ensemble.

**Fig. 2:**
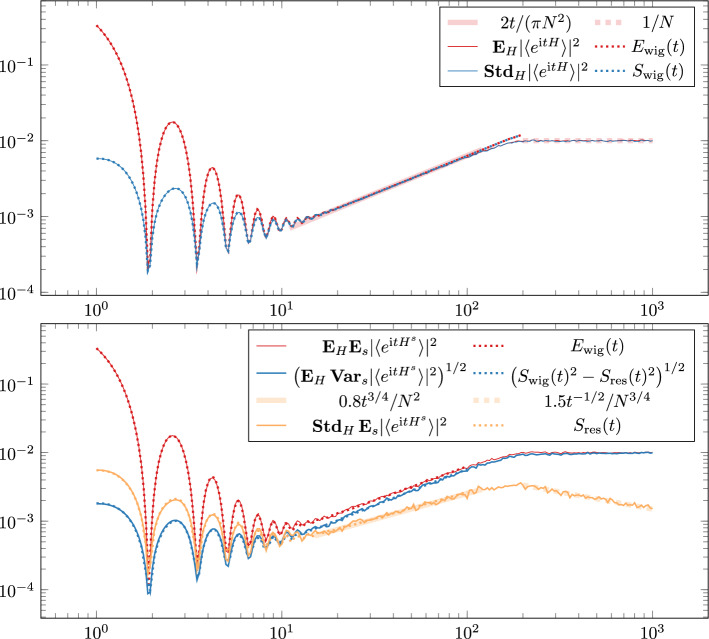
In the first plot we compare the empirical mean (red) and standard deviation (blue) of $|{\langle {e^{\textrm{i}t H}} \rangle } |^2$ obtained from sampling $10,000$ independent $100\times 100$ GUE matrices $H$ with our approximation (132.13). In the second plot we similarly compare the empirical mean (red) and variance (blue), with respect to *s*, obtained from sampling $500$ independent scalar random variables $s$ (from the uniform distribution on $S^1$) and $500$ independent $100\times 100$ GUE matrix pairs $H_1,H_2$, with the prediction (152.15). We also test the precision of the latter GUE-pair sampling by finding the empirical standard deviation (with respect to $H_1,H_2$) of the empirical mean of the monoparametric SFF (orange). We observe that for both ensembles our resolvent approximation seems valid for all $t<N$

#### Theorem 2.7

(SFF for the Wigner ensemble). For deterministic $t>0$ (possibly *N*-dependent) we have

2.8 $$\begin{aligned} \begin{aligned} {\textbf{E}}_H |{\langle {e^{\textrm{i}t H}} \rangle } |^2&= E_\textrm{wig}(t)(1+o(1)) \quad \textrm{for} \quad t\ll N^{5/11},\\ {{\,\mathrm{\textbf{Var}}\,}}_H |{\langle {e^{\textrm{i}t H}} \rangle } |^2&=S_\textrm{wig}(t)^2(1+o(1)) \quad \textrm{for} \quad t\ll N^{5/17}, \end{aligned} \end{aligned}$$

and we have the asymptotics

2.9 $$\begin{aligned} \begin{aligned} E_\textrm{wig}(t):=e(t)^2 + \frac{v^{ee}_{-,\kappa }(t)}{N^2}&\approx {\left\{ \begin{array}{ll} \frac{J_1(2t)^2}{t^2}, &{} 1\ll t\ll \sqrt{N} \\ \frac{2}{\pi } \frac{t}{N^2}, &{} \sqrt{N}\ll t\ll N,\end{array}\right. } \\ S_\textrm{wig}(t):=\biggl (\frac{v_{+,\kappa }^{ee}(t)^2+v_{-,\kappa }^{ee}(t)^2}{N^4} + 2e(t)^2\frac{v_{+,\kappa }^{ee}(t)+v_{-,\kappa }^{ee}(t)}{N^2}\biggr )^{1/2}&\approx {\left\{ \begin{array}{ll} \frac{2J_1(2t)}{\sqrt{\pi t}N}, &{} 1\ll t\ll \sqrt{N} \\ \frac{2}{\pi } \frac{t}{N^2}, &{} \sqrt{N}\ll t\ll N,\end{array}\right. } \end{aligned}\nonumber \\ \end{aligned}$$

where we set $e:=(1,0)\in S^1$.

This result shows that $S_\textrm{wig}(t)\ll E_\textrm{wig}(t)$ in the slope regime, $t\ll \sqrt{N}$, and $S_\textrm{wig}(t)\approx E_\textrm{wig}(t)$ in the ramp regime, $\sqrt{N}\ll t\ll N$ (see the first plot in Fig. 2). In particular, in the ramp regime the SFF is a non-negative random variable whose fluctuations are of the same size as its expectation. Thus the SFF is not self-averaging in the ramp regime, while it is self-averaging in the slope regime but only owing to the dominance of the function *e*(*t*) representing the edge effect. If one discounts the edge effect, i.e. artificially removes *e*(*t*), then $S_\textrm{wig}(t)\approx E_\textrm{wig}(t)$ would hold for all $1\ll t\ll N$, demonstrating the universal behavior of SFF in the entire slope-dip-ramp regime.

#### Theorem 2.8

(SFF for the monoparametric ensemble (2.2)). For $t>0$ (possibly *N*-dependent) we have

10 $$\begin{aligned} \begin{aligned} {\textbf{E}}_H{\textbf{E}}_s|{\langle {e^{\textrm{i}t H^s}} \rangle } |^2&= E_\textrm{wig}(t)(1+o(1))\quad \textrm{for} \quad t\ll N^{3/7} \\ {\textbf{E}}_H{{\,\mathrm{\textbf{Var}}\,}}_s|{\langle {e^{\textrm{i}t H^s}} \rangle } |^2&= \Bigl (S_\textrm{wig}(t)^2-S_\textrm{res}(t)^2\Bigr )(1+o(1))\quad \textrm{for}\quad t\ll N^{5/17}\\ {{\,\mathrm{\textbf{Var}}\,}}_H{\textbf{E}}_s|{\langle {e^{\textrm{i}t H^s}} \rangle } |^2&= S_\textrm{res}(t)^2 (1+o(1))\quad \textrm{for} \quad t\ll N^{1/4} \end{aligned} \end{aligned}$$

where the function

2.10 $$\begin{aligned} \begin{aligned} S_\textrm{res}(t)&:=\sqrt{{\textbf{E}}_s{\textbf{E}}_r \Bigl (\frac{v_{+,\kappa }^{sr}(t)^2+v_{-,\kappa }^{sr}(t)^2}{N^4} + 2e(t)^2\frac{v_{+,\kappa }^{sr}(t)+v_{-,\kappa }^{sr}(t)}{N^2}\Bigr )} \end{aligned} \end{aligned}$$

satisfies

2.11 $$\begin{aligned} \begin{aligned} S_\textrm{res}(t) \sim {\left\{ \begin{array}{ll} \frac{\psi (t)}{Nt^{5/4}}, &{} 1\ll t\ll \sqrt{N} \\ \frac{t^{3/4}}{N^2}, &{} \sqrt{N}\ll t\ll N,\end{array}\right. } \end{aligned} \end{aligned}$$

where $\psi (t)\sim 1$ is a positive function with some oscillation.

In particular, this result immediately shows the following concentration effect:

#### Corollary 2.9

For $1\ll t\ll N^{1/4}$ it holds that

2.12 $$\begin{aligned} {{\,\mathrm{\textbf{Var}}\,}}_H {\textbf{E}}_s|{\langle {e^{\textrm{i}t H^s}} \rangle } |^2 \lesssim \frac{1}{\sqrt{t}} {{\,\mathrm{\textbf{Var}}\,}}_H |{\langle {e^{\textrm{i}t H}} \rangle } |^2, \end{aligned}$$

i.e. averaging in $s$ reduces the size of the fluctuation of the SFF by a factor of $t^{-1/4}$.

Note that

13 $$\begin{aligned} S_\textrm{res}(t) \lesssim t^{-1/4} S_\textrm{wig}(t) \end{aligned}$$

both in the slope and ramp regimes showing that not only the expectation but also the variance of the SFF for the monoparametric ensemble coincide with those for the Wigner ensemble to leading order, hence they follow the universal pattern (red and blue curves in the second plot in Fig. 2). However, the dependence of ${\textbf{E}}_s [{{\,\mathrm{\textbf{SFF}}\,}}(t)]$ on the fixed Wigner matrix pair $(H_1, H_2)$ is still present, albeit to a lower order, expressed by the *residual standard deviation* $S_\textrm{res}(t)$ whose relative size decreases as $t^{-1/4}$ as *t* increases (orange curves in Fig. 2). It is quite remarkable that a single random mode *s* generates almost the entire randomness in the ensemble that is responsible for the universality of SFF. A similar phenomenon was manifested in the Wigner–Dyson universality proven in [15].

#### Remark 2.10

Based upon extensive numerics (see Fig. 2) we believe that (2.13), (2.15) and (2.18) hold up to any $t\ll N$, i.e. in the entire slope-dip-ramp regime and not only up to some fractional power of *N* as stated and proved rigorously. The proof for the entire regime $t\ll N$ is out of reach with the current technology based upon the multi-resolvent local law Lemma 3.3 whose error term does not trace the expected improvement due to different spectral parameters $z_1\ne z_2$. We expect that the entire ramp regime $t\ll N$ should be accessible by resolvent techniques if a sharp version of Lemma 3.3, tracing the gain from $z_1\ne z_2$, was available.

#### Remark 2.11

We stated Theorems 2.7 and 2.8 only for the first two moments but the CLT from Theorem 2.5 allows us to compute arbitrary moments ${\textbf{E}}|{\langle {e^{\textrm{i}t H}} \rangle } |^{2m}$ for the Wigner case and ${\textbf{E}}_s|{\langle {e^{\textrm{i}t H^s}} \rangle } |^{2m}$ for the monoparametric case (together with their concentration in the $(H_1, H_2)$-space), albeit with worsening error estimates. This would lead to rigorous results of the type (2.13) and (2.15) but for a shorter time scale $t\ll N^{c(m)}$ with some $c(m)>0$. However, in the spirit of Remark 2.10, we believe that $\langle {e^{itH^s}} \rangle$ can be approximated for any $t\ll N$, to leading order, by a family of complex Gaussians $\xi (t,s)$ of mean and variance

2.13 $$\begin{aligned} {\textbf{E}}\xi (t,s) = e(t), \quad {\textbf{E}}(\xi (t,s)-e(t))(\xi (t',s')-e(t')) = \frac{1}{N^2} v^{ss'}(e^{\textrm{i}t\cdot },e^{\textrm{i}t' \cdot }) \end{aligned}$$

with $v^{sr}$ from (2.7). Note that (202.20) also specifies the covariance of $\xi (t,s)$ and $\overline{\xi (t',s')}=\xi (-t',s')$ for different times.

The next lemma, to be proven in Sect. 3.2.4, provides explicit asymptotic formulas for $v^{ss}_\pm (t)$, in particular they imply the asymptotics in (2.14) together with $e(t)\sim t^{-3/2}$ (up to some oscillation due to the Bessel function) in the large *t* regime.

#### Lemma 2.12

For $s=r$ the functions $v^{ss}_\pm (t)$ appearing in (2.12) can be expressed as

14 $$\begin{aligned} \begin{aligned} v^{ss}_-(t)&=t^2 \Bigl [J_0(2t)^2 + 2J_1( 2 t)^2 - J_0( 2 t)J_2( 2 t)\Bigr ] = \frac{2 t}{\pi }-\frac{1+2\sin (4t)}{16 \pi t} + {{\mathcal {O}}}({t^{-2}})\\ v^{ss}_+(t)&=- t J_0(2t) J_1(2t) =\frac{\cos (4 t)}{2 \pi }-\frac{2+\sin (4 t)}{16 \pi t}+ {{\mathcal {O}}}({t^{-2}}). \end{aligned} \end{aligned}$$

The relation in (2.17) requires a stationary phase calculation that will be done separately in Sect. 5.

### Implications for sampling

Determining the standard deviation of $|{\langle {e^{\textrm{i}tH}} \rangle } |^2$ is important for numerical testing of (2.13). By taking the empirical average ${\textbf{E}}_H^n$ of $n$ independent Wigner matrices we may approximate the true expectation ${\textbf{E}}_H|{\langle {e^{\textrm{i}tH}} \rangle } |^2$ at a speed

**Fig. 3:**
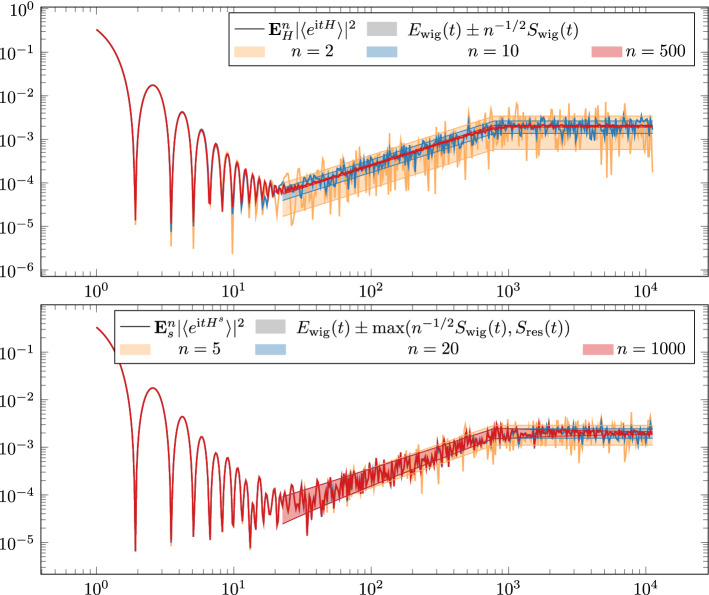
In the first plot we show the empirical mean of $|{\langle {e^{\textrm{i}tH}} \rangle } |^2$ for $k$ independent GUE matrices $H$. As expected the standard deviation of the sample average fluctuates within a strip of width $n^{-1/2}{{\,\mathrm{\textbf{Std}}\,}}_H|{\langle {e^{\textrm{i}t H}} \rangle } |^2$, in particular the sample average exactly reproduces the mean if $n\rightarrow \infty$. In the second plot we show the empirical mean of $|{\langle {e^{\textrm{i}tH^s}} \rangle } |^2$ for $k$ independently sampled scalar random variables $s$ for a fixed GUE matrix pair $H_1,H_2$. We observe that while the sample mean approximates the true mean ${\textbf{E}}_s$ increasingly well as $n\rightarrow \infty$, the latter is still dependent on the chosen realisation of $H_1,H_2$. Thus the empirical mean fluctuates in a strip of width $\max (n^{-1/2}S_\textrm{wig}(t),S_\textrm{res}(t))$ around the doubly averaged ${\textbf{E}}_H{\textbf{E}}_s|{\langle {e^{\textrm{i}tH^s}} \rangle } |^2$

2.14 $$\begin{aligned} {\textbf{E}}_H^n |{\langle {e^{\textrm{i}tH}} \rangle } |^2 = {\textbf{E}}_H |{\langle {e^{\textrm{i}tH}} \rangle } |^2 + \Omega \Big ( n^{-1/2}{{\,\mathrm{\textbf{Std}}\,}}_H |{\langle {e^{\textrm{i}tH}} \rangle } |^2\Big )=E_\textrm{wig}(t)+\Omega (n^{-1/2}S_\textrm{wig}(t)), \end{aligned}$$

c.f. the top of Fig. 3. Here $\Omega (\cdots )$ indicates an oscillatory error term of the given size. In the *ramp regime* the fluctuation of ${\textbf{E}}_H^n |{\langle {e^{\textrm{i}tH}} \rangle } |^2$ thus scales like $t/(\sqrt{n}N^{2})$ using (2.14). In particular, this fluctuation vanishes as the sample size *n* goes to infinite, hence the statistics via sampling to test (2.13) is *consistent*.

In contrast, for the monoparametric ensemble, by taking the empirical average of $n$ copies of $s$ we naturally have

15 $$\begin{aligned} {\textbf{E}}_s^n |{\langle {e^{\textrm{i}t H^s}} \rangle } |^2 = {\textbf{E}}_s |{\langle {e^{\textrm{i}t H^s}} \rangle } |^2 + \Omega \Big ( k^{-1/2} S_\textrm{wig}(t)\Big ). \end{aligned}$$

Replacing the first term by its expectation plus its fluctuation in the *H*-probability space, we also get

2.15 $$\begin{aligned} {\textbf{E}}_s^n |{\langle {e^{\textrm{i}t H^s}} \rangle } |^2 = {\textbf{E}}_H{\textbf{E}}_s |{\langle {e^{\textrm{i}t H^s}} \rangle } |^2 + \Omega \Bigl (\max \bigl (n^{-1/2}S_\textrm{wig}(t),S_\textrm{res}(t)\bigr )\Bigr ), \end{aligned}$$

where the error term contains both standard deviations and satisfies

16 $$\begin{aligned} \max \bigl (n^{-1/2}S_\textrm{wig}(t),S_\textrm{res}(t)\bigr ) \sim {\left\{ \begin{array}{ll} \frac{1}{Nt}\max \{ \frac{1}{\sqrt{n}},\frac{1}{t^{1/4}}\}, &{} 1\ll t\ll \sqrt{N} \\ \frac{t}{N^2}\max \{ \frac{1}{\sqrt{n}},\frac{1}{t^{1/4}}\} &{} \sqrt{N}\ll t\ll N,\end{array}\right. } \end{aligned}$$

due to (2.15) and (2.17). In particular, both in the slope and in the ramp regimes the size of the fluctuation of ${\textbf{E}}_s^n|{\langle {e^{\textrm{i}t H^s}} \rangle } |^2$

does not vanish even as the number of samples goes to infinity, $n\rightarrow \infty$, hence the statistics is *not consistent*, c.f. the bottom of Fig. 3. However, this lack of consistency, expressed by $S_\textrm{res}(t)$ is still negligible compared with the leading first term in (2.24) by a factor $t^{-1/4}\ll 1$ in the large *t* regime, see (2.19). We recall that mathematically rigorously we can prove all these facts only for $t\ll N^{1/4}$, i.e. well before the dip time, but the numerical tests leave no doubt on their validity in the entire regime $1\ll t\ll N$.

### Extensions

Beside the Wigner ensemble, we formulated our main results on SFF for the normalized monoparametric model in Theorem 2.8. We chose this model for definiteness, but our approach applies to the multi-parametric as well as to the un-normalised models introduced in Remark 2.4. Here we explain the modified results for these natural generalisations.

First, for the multi-parametric normalised model, $H^s=s_1H_1+\cdots + s_kH_k$ with $k-1$ effective parameters $s\in S^{k-1}$, Theorem 2.8 holds true verbatim modulo different sizes for the residual standard deviation $S_\textrm{res}(t)$. In fact, we have

2.16 $$\begin{aligned} S_\textrm{res}(t) \lesssim t^{-\frac{1}{2}+\frac{1}{4}(3-k)_+} S_\textrm{wig}(t), \end{aligned}$$

see (5.4) later, hence $S_\textrm{res}(t)$ becomes less relevant compared with $S_\textrm{wig}(t)$ for larger $k>2$, see (2.19). Consequently, the upper bounds on the times of proven validity in (152.15) slightly improve but they still remain below the dip time and we omit the precise formulas. We note that the $t$-power in (2.26) is not optimal for $k\ge 3$. A refined stationary phase estimate could be used to improve the estimate but we refrain from doing so since our primary interest is the mono-parametric model with few degrees of freedom.

Second, for the un-normalised model $H^s=s_1H_1+ s_2H_2$ with two effective parameters $s\in {\textbf{R}}^2$, Theorem 2.8 also holds true modulo some minor changes. More precisely, (152.15) becomes

17 $$\begin{aligned} \begin{aligned} {\textbf{E}}_H{\textbf{E}}_s|{\langle {e^{\textrm{i}t H^s}} \rangle } |^2&= {\textbf{E}}_s E_\textrm{wig}(\Vert s\Vert _2t)(1+o(1))\quad \textrm{for} \quad t\ll N^{3/7} \\ {\textbf{E}}_H{{\,\mathrm{\textbf{Var}}\,}}_s|{\langle {e^{\textrm{i}t H^s}} \rangle } |^2&= \Bigl ({\textbf{E}}_s S_\textrm{wig}(\Vert s\Vert _2t)^2-\widetilde{S}_\textrm{res}(t)^2\Bigr )(1+o(1))\quad \textrm{for}\quad t\ll N^{5/17}\\ {{\,\mathrm{\textbf{Var}}\,}}_H{\textbf{E}}_s|{\langle {e^{\textrm{i}t H^s}} \rangle } |^2&= \widetilde{S}_\textrm{res}(t)^2 (1+o(1))\quad \textrm{for} \quad t\ll N^{1/7}, \end{aligned} \end{aligned}$$

with $\widetilde{S}_\textrm{res}$ obtained from replacing $v^{sr}$ by $\widetilde{v}^{sr}$ from Remark 2.6 in (2.12). For $\widetilde{S}_\textrm{res}(t)$ a stationary phase calculation gives the modified

2.17 $$\begin{aligned} \widetilde{S}_\textrm{res}(t) \sim {\left\{ \begin{array}{ll} \frac{\psi (t)}{Nt^{7/4}}, &{} 1\ll t\ll \sqrt{N} \\ \frac{t^{1/4}}{N^2}, &{} \sqrt{N}\ll t\ll N, \end{array}\right. } \end{aligned}$$

assuming that *s* has an absolutely continuous distribution with a differentiable, compactly supported density $\rho$ on ${\textbf{R}}^2$ with $\rho (0)=0$. We will not prove the relation in these formulas in this paper, we only show how to obtain the necessary upper bound on them at the end of Sect. 5.

Note that now

18 $$\begin{aligned} \widetilde{S}_\textrm{res}(t)\lesssim t^{-3/4}{\textbf{E}}_s S_\textrm{wig}(\Vert s\Vert _2t), \end{aligned}$$

i.e. the fluctuation due to the residual randomness of $(H_1, H_2)$ after taking the expectation in *s* remains negligible, in fact it is reduced compared with the normalised case (2.19). As a consequence $t^{1/4}$ in (2.25) is replaced by $t^{3/4}$.

Analogous results hold for the most general multi-parametric un-normalised model as well as to the mono-parametric inhomogeneous model $H^s=H_1+sH_2$, $s\in {\textbf{R}}$. We omit their precise formulation, the key point is that the analogue of (2.27) hold in all cases with a residual standard deviation $\widetilde{S}_\textrm{res}(t)$ being smaller than the leading term $S_\textrm{wig}(t)$ by a polynomial factor in *t* (e.g. by $t^{-1/2}$ for $H^s=H_1+sH_2$). This guarantees that the universality of SFF holds for all these models. Table 1 summarizes the decay exponents of our main parametric models.

**Table 1:** For our three main parametric models the following table lists the size of the residual fluctuation compared to the fluctuation of the Wigner-SFF

Quenched parametric model	Randomness	
$s_1 H_1 + s_2 H_2$	$(s_1, s_2)\in S^1$	$S_\textrm{res}(t) \lesssim t^{-1/4} S_\textrm{wig}(t)$
$H_1 + s H_2$	$s\in {\textbf{R}}$	$S_\textrm{res}(t) \lesssim t^{-1/2} S_\textrm{wig}(t)$
$s_1 H_1 + s_2 H_2$	$(s_1, s_2)\in {\textbf{R}}^2$	$S_\textrm{res}(t) \lesssim t^{-3/4} S_\textrm{wig}(t)$

### Outline

The rest of the paper is organised as follows. In Sect. 3 we outline the resolvent method and explain how via the Helffer–Sjöstrand representation a resolvent CLT implies the CLT for the linear statistics $\sum f(\lambda _i)$ of arbitrary test functions $f$ from which our main results Theorems 2.5–2.8 follow. In Sect. 4 we present the proof of the resolvent CLT, while in Sect. 5 we conclude the proof of the asympotics (172.17) via a stationary phase argument.

## Resolvent Method

Let *H* be a Wigner matrix^5^ and $G(z):= (H-z)^{-1}$ its resolvent with a spectral parameter $z\in {\textbf{C}}{\setminus } {\textbf{R}}$. Define $m_\textrm{sc}(z)$, the Stieltjes transform of the semicircle law:

2.18 $$\begin{aligned} m(z)=m_\textrm{sc}(z):=\int _{\textbf{R}}\frac{\rho _\textrm{sc}(x)}{x-z} {\text {d}}\!{}x, \quad \rho _\textrm{sc}(x):= \frac{\sqrt{(4-x^2)_+}}{2\pi }. \end{aligned}$$

The *local law* for a single resolvent states that the diagonal matrix $m(z)\cdot I$ well approximates the random resolvent *G*(*z*) in the following sense (see e.g. [5, 20, 35]):

2.19 $$\begin{aligned} |\langle {(G(z)-m(z))A} \rangle | \lesssim N^\xi \frac{\Vert A\Vert }{N\eta }, \qquad \langle {\varvec{x}}, (G(z)-m(z)) {\varvec{y}}\rangle \lesssim N^\xi \frac{\Vert {\varvec{x}}\Vert \Vert {\varvec{y}}\Vert }{\sqrt{N\eta }} \end{aligned}$$

with $\eta = |\Im z|$, for any fixed deterministic matrix *A* and deterministic vectors ${\varvec{x}}, {\varvec{y}}$. The first bound is called *averaged* local law, while the second one is the *isotropic* local law. The bounds (3.2) are understood in very high probability for any fixed $\xi >0$.

The Helffer–Sjöstrand formula

20 $$\begin{aligned} \langle {f(H)} \rangle =\frac{2}{\pi }\int _{\textbf{C}}\partial _{{\overline{z}}}f_{\textbf{C}}(z) \langle {G(z)} \rangle {\text {d}}\!{}^2 z, \end{aligned}$$

with $z=x+\textrm{i}\eta$ and ${\text {d}}\!{}^2 z:={\text {d}}\!{}\eta {\text {d}}\!{}x$, expresses the linear statistics of arbitrary functions as an integral of the resolvent $G(z)$ and the almost-analytic extension

2.20 $$\begin{aligned} f_{\textbf{C}}(z)=f_{\textbf{C}}(x+\textrm{i}\eta ):=\big [f(x)+\textrm{i}\eta \partial _x f(x)\big ]\chi (\tau \eta ), \end{aligned}$$

of $f$. Here the free parameter $\tau \in {\textbf{R}}$ is chosen such that $N^{-1}\ll \tau ^{-1}\lesssim 1$, and $\chi$ a smooth cut-off equal to 1 on $[-5,5]$ and equal to 0 on $[-10,10]^c$. The same $\tau$ was used to define the weighted $H^2$-norm (2.4) and eventually we will optimize its value, a procedure that improves the standard error terms in the CLT. By (3.2) it follows that

21 $$\begin{aligned} \langle {f(H)} \rangle = \frac{2}{\pi } \int _{\textbf{C}}\partial _{{\overline{z}}}f_{\textbf{C}}(z) m(z){\text {d}}\!{}^2 z + {{\mathcal {O}}}({*}){N^\xi \frac{\Vert f\Vert _{H^2}}{N}} = \int _{-2}^2 \rho _\textrm{sc}(x) f(x){\text {d}}\!{}x + {{\mathcal {O}}}({*}){N^\xi \frac{\Vert f\Vert _{H^2}}{N}}. \end{aligned}$$

In order to compute the fluctuation in (3.5) via (3.3) we need to understand the correlation between $\langle {G(z)} \rangle ,\langle {G(z')} \rangle$ for two different spectral parameters $z,z'$ which turns out to be given by

2.21 $$\begin{aligned} {{\,\mathrm{\textbf{Cov}}\,}}(\langle {G(z)} \rangle ,\langle {G(z')} \rangle ) \approx \frac{1}{N^2}\frac{\langle {G(z)^2} \rangle \langle {G(z')^2} \rangle \langle {G(z)G(z')} \rangle (1+\langle {G(z)G(z')} \rangle )}{\langle {G(z)} \rangle \langle {G(z')} \rangle }, \end{aligned}$$

modulo some additional contribution from non-Gaussian fourth cumulant, see (83.8) for the final statement. While $G(z)\approx m(z)$, in general it is not true that $G(z) G(z') \approx m(z)m(z')$ since (3.2) allows only deterministic test matrices multiplying *G*. Nevertheless $G(z) G(z')$ is still approximable by a deterministic object:

2.22 $$\begin{aligned} G(z) G(z') \approx \frac{m(z) m(z')}{1- m(z) m(z')}. \end{aligned}$$

Statements of the form (3.7) with an appropriate error term are called *multi-resolvent local laws*.

We will apply this theory to the product of the resolvents $G^s$ of $H^s=s_1H_1+s_2H_2$ for two different parameters *s*, see the corresponding local law on $\langle {G^s G^r} \rangle$ in (3.11) later. Even though $H_1$ and $H_2$ as well as *s* and *r* are independent, the common $(H_1, H_2)$ ingredients in $H^s$ and $H^r$ introduce a nontrivial correlation between these matrices. We therefore need to extend CLT for resolvents via multi-resolvent local laws to this parametric situation.

### Resolvent CLT

The main technical result of the present paper is the following Central Limit Theorem for product of resolvents of the random matrix $H^s:=s_1H_1+s_2H_2$ with $s=(s_1,s_2)\in S^1$.

#### Proposition 3.1

Fix $\epsilon >0$, $p\in {\textbf{N}}$, $s^1,\dots ,s^p\in S^1$, $z_1,\dots ,z_p\in {\textbf{C}}{\setminus }{\textbf{R}}$, and define $G_i:=(H^{s^i}-z_i)^{-1}$. Then for any arbitrary small $\xi >0$ and $\eta _*\ge N^{-1+\epsilon }$ it holds

2.23 $$\begin{aligned} {\textbf{E}}_H\prod _{i\in [p]}\langle {G_i-{\textbf{E}}_H G_i} \rangle =\frac{1}{N^p}\sum _{P\in \textrm{Pair}([p])}\prod _{(i,j)\in P} V_{ij}+{\mathcal {O}}\left( N^\xi \Psi _p\left( \frac{1}{L^{1/2}} +\frac{1}{N\eta _*^2}+\frac{1}{N^2\eta _*^4}\right) \right) , \quad \Psi _p:=\prod _{i\in [p]}\frac{1}{N|\eta _i|}. \nonumber \\ \end{aligned}$$

Here $$\eta _i:=\Im z_i$$, $$\eta _*:=\min _i|{\eta _i} |$$, $$L:=\min _i (N\eta _i\rho _i)$$, and

2.24 $$\begin{aligned} V_{ij}:=-\frac{2}{\beta }\partial _{z_i}\partial _{z_j}\log \biggl (1-\langle {s^i,s^j} \rangle m_im_j\biggr )-\langle {s^i\odot s^i,s^j\odot s^j} \rangle \kappa _4(m_i^2)'(m_j^2)', \end{aligned}$$

where $m_i:=m_{\textrm{sc}}(z_i)$, and $\kappa _4:=N^2{\textbf{E}}|h_{12}|^4-1-2/\beta$. Additionally, for the expectation we have

2.25 $$\begin{aligned} {\textbf{E}}_H\langle {G_i} \rangle =m_i+\frac{\kappa _4}{N}\Vert s\Vert _4^4 m_i'm_i^3+\varvec{1}(\beta =1)\frac{1}{N}\frac{m_im_i'}{1-m_i^2} +{\mathcal {O}}\left( N^\xi \frac{\sqrt{\rho _i}}{(N\eta _i)^{3/2}}\right) , \end{aligned}$$

with $$\rho _i:=\pi ^{-1}|\Im m_i|$$.

#### Remark 3.2

For Wigner matrices, i.e. for $s^1=\dots =s^p=(1,0)$, the error term in (3.8) is given by $\Psi L^{-1/2}$, as a consequence of the fact that the error terms in the first and second line of (3.11) are replaced by $(N\eta _1\eta _2)^{-1}$ and $(N\eta _1\eta _2^2)^{-1}$, respectively (see e.g. [16, Remark 3.5]).

We point out that similar resolvent CLT have often been used as a basic input to prove CLT for linear eigenvalue statistics of both Hermitian and non-Hermitian matrices down to optimal mesoscopic scales (see e.g. [10, 11, 14, 28–30, 36–38]). The main novelty here is to extend the resolvent CLT to the monoparametric ensemble.

Along the proof of Proposition 3.1 we establish the following multi-resolvent local laws.

#### Lemma 3.3

For $G_i=G^{s^i}(z_i)$ we have the two- and three-resolvent local laws

2.26 $$\begin{aligned} \begin{aligned} |{\langle {G_1G_2} \rangle -\frac{m_1m_2}{1-\langle {s^1,s^2} \rangle m_1m_2}} |&\lesssim \frac{N^\xi }{N|\eta _1\eta _2|^{3/2}}\\ |{\langle {G_1G_2^2} \rangle -\frac{m_1m_2'}{(1-\langle {s^1,s^2} \rangle m_1m_2)^2}} |&\lesssim \frac{N^\xi }{N|\eta _1||\eta _2|\eta _*^2}+\frac{1}{N^2|\eta _1\eta _2|^3}, \end{aligned} \end{aligned}$$

where $m_i=m_\textrm{sc}(z_i)$, with very high probability for any fixed $\xi ,\epsilon >0$ and $|\Im z_i|\ge N^{-1+\epsilon }$.

The proofs of Proposition 3.1 and Lemma 3.3 will be presented in Sect. 4. In these proofs we will often use the standard *cumulant expansion* (see [8, 30, 34] in the random matrix context):

2.27 $$\begin{aligned} {\textbf{E}}_H h_{ab}f(H)=\frac{1}{N}{\textbf{E}}_H\partial _{ba} f(H)+\sum _{k=2}^R\sum _{q+q'=k} \frac{\kappa _{ab}^{q+1,q'}}{N^{(k+1)/2}}{\textbf{E}}_H \partial _{ab}^q\partial _{ba}^{q'}f(H)+\Omega _R. \end{aligned}$$

Here $\partial _{ab}$ denotes the directional derivative $\partial _{h_{ab}}$, the first term in the rhs. represents the second order (Gaussian) contribution, while the sum in (3.12) represents the non-Gaussian contribution with $\kappa ^{p,q}_{ab}$ denoting the joint cumulant of *p* copies of $N^{1/2}h_{ab}$ and *q* copies of $N^{1/2}\overline{h_{ab}}$. The cumulant expansion is typically truncated at a high (*N*-independent) level *R* with an error term $\Omega _R$ that is negligible. To see this, note that in our applications *f* will be a product of resolvents at spectral parameters $z_i$ with $\eta _* = \min |\Im z_i|\gg 1/N$ hence derivatives of *f* remain bounded with very high probability by the isotropic local law (3.2) thus the tail of the series (3.12) decays as $N^{-(k+1)/2}$.

### Proof of Theorem 2.5

The proof of Theorem 2.5 is divided into three steps: (i) computation of the expectation, (ii) computation of the variance, (iii) proof of Wick Theorem. The expectation is computed in Sect. 3.2.1, while the Wick Theorem and the explicit computation of the variance are proven in Sect. 3.2.2.

#### Expectation

Using the bound

2.28 $$\begin{aligned} \big |\partial _{{\overline{z}}}f_{\textbf{C}}\big |\lesssim \eta |f''|+\tau |\chi '|\big [|f|+\textrm{i}\eta |f'|\big ], \end{aligned}$$

and $|\langle {G^s-m} \rangle |\lesssim N^\xi (N\eta )^{-1}$ by (3.2), with $m=m_{\textrm{sc}}$, we conclude that

2.29 $$\begin{aligned} {\textbf{E}}_H\langle {f(H^s)} \rangle =\int _{\textbf{R}}\int _{|\eta |\ge \eta _0}\partial _{{\overline{z}}}f_{\textbf{C}}(z){\textbf{E}}_H\langle {G^s(z)} \rangle {\text {d}}\!{}\eta {\text {d}}\!{}x+{\mathcal {O}}\left( \frac{N^\xi \eta _0\Vert f\Vert _{H^2}}{N}+N^\xi \eta _0^2\Vert f\Vert _{H^2}\right) , \end{aligned}$$

for any $N^{-1}\ll \eta _0\ll \tau ^{-1}$. Note that we chose $\eta _0\gg N^{-1}$ in order to use Proposition 3.1.

Plugging (3.10) into (3.14), and using (3.13) to estimate the error term, we get that

3.1 $$\begin{aligned} \begin{aligned}&{\textbf{E}}_H\langle {f(H^s)} \rangle =\int _{\textbf{R}}\int _{|\eta |\ge \eta _0}\partial _{{\overline{z}}}f_{\textbf{C}}(z)\Big [m +\frac{\kappa _4}{N}\Vert s\Vert _4^4m'm^3+\varvec{1}(\beta =1)\frac{1}{N}\frac{mm'}{1-m^2}\Big ]{\text {d}}\!{}\eta {\text {d}}\!{}x\\&\quad +{\mathcal {O}}\left( \frac{N^\xi \eta _0\Vert f\Vert _{H^2}}{N}+N^\xi \eta _0^2\Vert f\Vert _{H^2}+\frac{N^\xi \Vert f\Vert _{H^2}}{N^{3/2}\tau ^{1/2}}+\frac{N^\xi \tau ^{3/2}\Vert f\Vert _\infty }{N^{3/2}}+\frac{N^\xi \tau ^{1/2}\Vert f\Vert _{H^1}}{N^{3/2}}\right) \\&\quad =\int _{\textbf{R}}\int _{|\eta |\ge \eta _0}\partial _{{\overline{z}}}f_{\textbf{C}}(z)\Big [m+\frac{\kappa _4}{N} \Vert s\Vert _4^4m'm^3+\varvec{1}(\beta =1)\frac{1}{N}\frac{mm'}{1-m^2}\Big ]{\text {d}}\!{}\eta {\text {d}}\!{}x+{\mathcal {O}}\left( \frac{N^\xi \Vert f\Vert _{\tau }}{N^{3/2}\tau ^{1/2}}\right) , \end{aligned} \end{aligned}$$

where to go to the last line we chose $\eta _0\sim N^{-1+\epsilon }$, for some very small $\epsilon >0$, and we used the norm $\Vert f\Vert _{\tau }$ defined in (2.4).

Adding back the regime $|\eta |< \eta _0$ at the price of a negligible error smaller than the one in (3.15), by explicit computations (exactly as in [13, Section D.1]) in the leading term of (3.15), we conclude

3.2 $$\begin{aligned} \begin{aligned} {\textbf{E}}_H \langle {f(H^s)} \rangle&= \int _{-2}^2 \rho _\textrm{sc}(x) f(x){\text {d}}\!{}x + \frac{\kappa _4}{2N}\Vert s\Vert _4^4 \int _{-2}^2 \frac{x^4-4x^2+2}{\pi \sqrt{4-x^2}}f(x){\text {d}}\!{}x \\&\quad +\varvec{1}(\beta =1)\left[ \frac{f(2)+f(-2)}{4N}-\frac{1}{2\pi N}\int _{-2}^2 \frac{f(x)}{\sqrt{4-x^2}}\,{\text {d}}\!{}x\right] +{\mathcal {O}}\left( \frac{N^\xi \Vert f\Vert _{\tau }}{N^{3/2}\tau ^{1/2}}\right) . \end{aligned} \end{aligned}$$

#### Second moment and Wick theorem

Define

3.3 $$\begin{aligned} L_N(f,s):=N[\langle {f(H^s)} \rangle -{\textbf{E}}_H \langle {f(H^s)} \rangle ], \end{aligned}$$

then in this section, using Proposition 3.1, we compute the leading order term of ${\textbf{E}}_H L_N(f_1,s^1)L_N(f_2,s^2)$. More precisely, by (3.8) for $p=2$, and using (3.13) to estimate the error term, it follows that

3.4 $$\begin{aligned} \begin{aligned}&{\textbf{E}}_H L_N(f_1,s^1)L_N(f_2,s^2)\\&=\iint _{\textbf{R}}\iint _{|\eta _1|,|\eta _2|\ge \eta _0}\partial _{\overline{z_1}}f_{\textbf{C}}(z_1)\partial _{\overline{z_2}}f_{\textbf{C}}(z_2) V_{12} \\&\quad +{\mathcal {O}}\Bigg (N^\xi \eta _0(\Vert f_1\Vert _{H^2}\Vert f_2\Vert _\infty +\Vert f_2\Vert _{H^2}\Vert f_1\Vert _\infty )+\frac{N^\xi \Vert f_1\Vert _\tau \Vert f_2\Vert _\tau }{N^{1/2}\tau ^{3/2}}+\frac{\Vert f_1\Vert _{H^2}\Vert f_2\Vert _{H^2}}{N^{1-\xi }\eta _0\tau } \left( 1+\frac{1}{N\eta _0^2}\right) \\&\quad \qquad +\frac{(\Vert f_1\Vert _{H^2}(\tau ^2\Vert f_2\Vert _\infty +\tau \Vert f_2\Vert _{H^1}) +\Vert f_2\Vert _{H^2}(\tau ^2\Vert f_1\Vert _\infty +\tau \Vert f_1\Vert _{H^1}))}{N^{1-\xi }\eta _0\tau } \left( 1+\frac{1}{N\eta _0^2}\right) \\&\quad \qquad +\frac{(\tau ^2\Vert f_1\Vert _\infty +\tau \Vert f_1\Vert _{H^1})(\tau ^2\Vert f_2\Vert _\infty +\tau \Vert f_2\Vert _{H^1})}{N}\left( 1+\frac{\tau ^2}{N^{1-2\epsilon }}\right) \Bigg )\\&=\iint _{\textbf{R}}\iint _{|\eta _1|,|\eta _2|\ge N^{-\epsilon }\tau ^{-1}}\partial _{\overline{z_1}} f_{\textbf{C}}(z_1)\partial _{\overline{z_2}}f_{\textbf{C}}(z_2) V_{12}\\&\quad +{\mathcal {O}}\left( N^\xi \Vert f_1\Vert _\tau \Vert f_2\Vert _\tau \left( \frac{N^\epsilon }{N}+\frac{N^{-\epsilon }}{\tau ^3}\right) \left( 1+\frac{\tau ^2}{N^{1-2\epsilon }}\right) \right) , \end{aligned} \end{aligned}$$

where to go to the last line we chose $\eta _0\sim N^{-\epsilon }\tau ^{-1}$, for any $\epsilon >0$, and $V_{12}$ is defined in (3.9). From (3.18), adding back the regimes $|\eta _i|< N^{-\epsilon }\tau ^{-1}$ at the price of an error smaller than the one in the last line of (3.18), we conclude (2.6) for $p=2$ by explicit computation in deterministic term as in [13, Section D.2].

We conclude this section with the computation of higher moments:

3.5 $$\begin{aligned} \begin{aligned} {\textbf{E}}_H\prod _{i\in [p]}L_N(f_i,s^i)&=\sum _{P\in \mathrm {Pair([p])}} \prod _{(i,j)\in P}\iint _{\textbf{R}}\iint _{|\eta _i|,|\eta _j|\ge N^{-\epsilon }}\partial _{\overline{z_i}}f_{\textbf{C}}(z_i)\partial _{\overline{z_j}}f_{\textbf{C}}(z_j) V_{ij}\\&\quad +{\mathcal {O}}\left( \left( \frac{N^\xi }{N^{1/2}\tau ^{3/2}} +\frac{N^\epsilon }{N}+\frac{N^{-\epsilon }}{\tau ^{2p-1}}\right) \left( 1+\frac{\tau ^2}{N^{1-2\epsilon }}\right) \prod _{i\in [p]}\Vert f_i\Vert _\tau \right) , \end{aligned} \end{aligned}$$

which concludes the proof of (2.6) for any $p\in {\textbf{N}}$, after adding back the regimes $|\eta _i|< N^{-\epsilon }\tau ^{-1}$ at the price of an error smaller than the one in the second line of (3.19).

#### Proof of Theorems 2.7 and 2.8

We just show how Theorem 2.7 follows by Theorem 2.5; the proof of Theorems 2.8 is completely analogous and so omitted. In particular, to make the presentation shorter we just show the details of the proof of the first equation in (2.13). Using Theorem 2.5 as an input, the proof of the second equation in (2.13) follows exactly in the same way.

First of all we write

3.6 $$\begin{aligned} {\textbf{E}}_H|\langle {e^{\textrm{i}t H}} \rangle |^2={\textbf{E}}_H\big |\langle {e^{\textrm{i}t H}} \rangle -{\textbf{E}}_H\langle {e^{\textrm{i}t H}} \rangle \big |^2+ \big |{\textbf{E}}_H\langle {e^{\textrm{i}t H}} \rangle \big |^2. \end{aligned}$$

Then, using (2.6) with $p=2$, $f_1(x)=e^{\textrm{i}tx}$, $f_2(x)=e^{-\textrm{i}tx}$, and $\tau =t$ to compute the leading order of the first term in (3.20), and (2.5) with $f(x)=e^{itx}$ to compute the leading order of the second term in (3.20), we conclude that

3.7 $$\begin{aligned} {\textbf{E}}_H|\langle {e^{\textrm{i}t H}} \rangle |^2=E_{\textrm{wig}}(t)+{\mathcal {O}}\left( \frac{1}{N^{3/2}}+\frac{t^{5/2}}{N^{5/2}}\right) , \end{aligned}$$

with $E_{\textrm{wig}}(t)$ defined in (2.14). Finally, using the asymptotics of $E_{\textrm{wig}}(t)$ in (2.14) we readily conclude that the error term in (3.21) is much smaller than the leading term $E_{\textrm{wig}}(t)$ as long as $t\ll N^{5/11}$.

#### Variance calculations when $s=r$ and the proof of Lemma 2.12

We note that (2.7) generalises the standard variance calculation yielding (1.7) to $s\ne r$. For the case $s=r$ the two formulas can be seen to be equivalent using the identity

8 $$\begin{aligned} \begin{aligned}&\frac{1}{2\pi ^2}\iint _{-2}^2 f'(x)g'(y) \log |{\frac{1-m_\textrm{sc}(x)\overline{m_\textrm{sc}(y)}}{1-m_\textrm{sc}(x)m_\textrm{sc}(y)}} |{\text {d}}\!{}x{\text {d}}\!{}y \\&\quad = \frac{1}{4\pi ^2} \iint _{-2}^2 \frac{f(x)-f(y)}{x-y}\frac{g(x)-g(y)}{x-y} \frac{4-xy}{\sqrt{4-x^2}\sqrt{4-y^2}}{\text {d}}\!{}x {\text {d}}\!{}y \end{aligned} \end{aligned}$$

that can be proven by integration by parts and using $(m_\textrm{sc}(x) + x)m_\textrm{sc}(x)=-1$ from the explicit form of $m_\textrm{sc}(x)$ from (3.1).

##### Proof of Lemma 2.12

Using (3.22) the functions $v^{ss}_\pm (t)$ appearing in (2.12) can be expressed as

3.8 $$\begin{aligned} \begin{aligned} v^{ss}_-(t)&=\frac{1}{\pi ^2}\int _{-1}^1\int _{-1}^1 \frac{1-x y}{ \sqrt{1-x^2} \sqrt{1-y^2} }\Bigl (\frac{\sin \left( t(x-y)\right) }{x-y}\Bigr )^2 {\text {d}}\!{}x{\text {d}}\!{}y\\&= \sum _{k=1}^\infty k J_{k}(2t)^2 = t^2 \Bigl [J_0(2t)^2 + 2J_1( 2 t)^2 - J_0( 2 t)J_2( 2 t)\Bigr ] \\&= \frac{2 t}{\pi }-\frac{1+2\sin (4t)}{16 \pi t} + {{\mathcal {O}}}({t^{-2}}) \end{aligned} \end{aligned}$$

and

9 $$\begin{aligned} \begin{aligned} v^{ss}_+(t)&=\frac{1}{4\pi ^2}\int _{-1}^1\int _{-1}^1 \frac{1-x y}{ \sqrt{1-x^2} \sqrt{1-y^2} }\Bigl (\frac{e^{2\textrm{i}tx} - e^{2\textrm{i}ty}}{x-y}\Bigr )^2 {\text {d}}\!{}x{\text {d}}\!{}y\\&=\sum _{k=1}^\infty (-1)^k k J_k(2t)^2 = - t J_0(2t) J_1(2t)\\&=\frac{\cos (4 t)}{2 \pi }-\frac{2+\sin (4 t)}{16 \pi t}+ {{\mathcal {O}}}({t^{-2}}) \end{aligned} \end{aligned}$$

where the series representations follow directly from [13, Remark 2.6] and the series evaluations follow from [50, V.§ 5.51(1)]. $\square$

## Central Limit Theorem for Resolvents

The proof of Proposition 3.1 is divided into three parts: in Sect. 4.1 we compute the subleading order correction to ${\textbf{E}}_H \langle {G_i} \rangle$, in Sect. 4.2 we explicitly compute the variance, and finally in Sect. 4.3 we prove a Wick Theorem. To keep our presentation simpler we only prove the CLT for resolvent in the complex case, the real case is completely analogous and so omitted (see e.g. [13, Section 4]).

### Computation of the expectation

For $G=G^s(z)$ we have

3.9 $$\begin{aligned} I =s_1\underline{H_1 G} + s_2 \underline{H_2 G} -\langle {G} \rangle G - zG, \quad \underline{H_i G}:=H_1 G+s_i\langle {G} \rangle G \end{aligned}$$

so that $G\approx m$ for the solution $m$ to the equation

10 $$\begin{aligned} -\frac{1}{m} = z + m, \qquad m(z)=m_\textrm{sc}(z). \end{aligned}$$

The fact that $G\approx m$ in averaged and isotropic sense follows from the single resolvent local law (3.2). This is a consequence of the fact that the term $\underline{H_i G}$ in (4.1) is designed in such a way ${\textbf{E}}\underline{H_i G}\approx 0$ in averaged and isotropic sense. In fact, for Gaussian ensembles ${\textbf{E}}\underline{H_i G}=0$ and the deviation from zero for general ensembles is a lower order effect due to non-vanishing of higher order cumulants of the entry distribution. From (4.1) and (4.2) we obtain

3.10 $$\begin{aligned} (1-m^2\langle {\cdot } \rangle )[G-m] = - m(s_1\underline{H_1 G}+s_2\underline{H_2G}) + m \langle {G-m} \rangle (G-m). \end{aligned}$$

Additionally, we define $\rho (z):=\pi ^{-1}|\Im m(z)|$. For simplicity of notation from now on we assume that $\Im z>0$. We remark that by $1-m^2\langle {\cdot } \rangle$ in the lhs. of (4.3) we denote the operator acting on matrices $R\in {\textbf{C}}^{N\times N}$ as $(1-m^2\langle {\cdot } \rangle )[R]=R-m^2\langle {R} \rangle$.

We then start computing:

11 $$\begin{aligned} {\textbf{E}}_H \langle {G-m} \rangle =-\frac{m'}{m}{\textbf{E}}_H\langle {s_1\underline{H_1 G}+s_2\underline{H_2G}} \rangle +{\mathcal {O}}\left( \frac{N^\xi }{N^2\eta ^2\rho }\right) , \end{aligned}$$

for any small $\xi >0$, where we used that $|1-m^2|\gtrsim \rho$, that $m'=m^2/(1-m^2)$, and that $|\langle {G-m} \rangle |\lesssim N^\xi (N\eta )^{-1}$ by (3.2). Then using cumulant expansion (see (3.12), ignoring the truncation error) we claim (and prove below) that

3.11 $$\begin{aligned} \begin{aligned} {\textbf{E}}_H\langle {s_1^1\underline{H_1 G}+s_2^1\underline{H_2G}} \rangle&={\textbf{E}}_H\frac{1}{N}\sum _{k\ge 2}\sum _{ab}\sum _{{\varvec{\alpha }}\in \{ab,ba\}^k}\left( \frac{\kappa ^{(1)}(ab,{\varvec{\alpha }})}{k!}s_1\partial _{\varvec{\alpha }}^{(1)}+\frac{\kappa ^{(2)}(ab,{\varvec{\alpha }})}{k!}s_2\partial _{\varvec{\alpha }}^{(2)}\right) G_{ba} \\&=\frac{\kappa _4}{N}\Vert s\Vert _4^4 m^4+{\mathcal {O}}\left( \frac{N^\xi \rho ^{3/2}}{N^{3/2}\eta ^{1/2}}+\frac{N^\xi \rho ^{3/2}}{N^2\eta ^{3/2}}\right) , \end{aligned} \end{aligned}$$

where $\kappa ^{(i)}(ab,{\varvec{\alpha }})$ denotes the joint cumulant of the random variables $h_{ab}^i$, $h_{\alpha _1}^i, \dots , h_{\alpha _k}^i$, and $\partial _{\varvec{\alpha }}^{(i)}:=\partial _{\alpha _1}^{(i)}\cdots \partial _{\alpha _k}^{(i)}$, with $i=1,2$, where $\partial _{\alpha _j}^{(i)}$ denotes the directional derivative in the direction $h_{\alpha _j}^i$. Here $h_{\alpha _j}^i$ are the entries of $H_i$. Combining (4.5) with (4.4) we obtain exactly the expansion in (3.10) (recall that here we only present the proof in the complex case, the real case being completely analogous).

#### Proof of the second equality in (4.5)

First of all we recall that by (2.1) it follows the bound $|\kappa ^{(i)}(ab,{\varvec{\alpha }})|\lesssim N^{-(k+1)/2}$, with $i=1,2$.

We start with $k=2$. In this case we can neglect the summation when $a=b$ since it gives a contribution $N^{-3/2}$. Hence we can assume that $a\ne b$. In this case we have the bounds

3.12 $$\begin{aligned} N^{-5/2}\left| \sum _{a\ne b} G_{ab}^3\right| \lesssim \frac{N^\xi \rho ^{3/2}}{N^2\eta ^{3/2}}, \qquad N^{-5/2}\left| \sum _{a\ne b} G_{aa}G_{bb}G_{ab}\right| \lesssim \frac{N^\xi }{N^{3/2}}+\frac{N^\xi \rho ^{3/2}}{N^2\eta ^{3/2}}, \end{aligned}$$

with very high probability. The first bound in (4.6) follows from the isotropic law in (3.2). The second bound in (4.6) follows by writing $G=m+(G-m)$ and using the isotropic resummation

3.13 $$\begin{aligned} \sum _{ab} (G-m)_{aa}G_{ab}=\sum _a\langle {{\varvec{e}}_a,G \varvec{1}} \rangle , \end{aligned}$$

with ${\varvec{e}}_a\in {\textbf{R}}^N$ the unit vector in the *a*-direction and $\varvec{1}:=(1,\dots ,1)\in {\textbf{R}}^N$.

For $k=3$ whenever there are at least two off-diagonal *G*’s we get a bound $N^{-2}\eta ^{-1}\rho$. The only way to get only diagonal *G*’s is that ${\varvec{\alpha }}$ is one of (*ab*, *ba*, *ba*), (*ba*, *ab*, *ba*), (*ba*, *ba*, *ab*); in this case $\kappa ^{(i)}(ab,{\varvec{\alpha }})=\kappa _4/N^2$, with $\kappa _4:=\kappa ^{(i)}(ab,ba,ab,ba)$. For these terms we have (see [13, Lemma 4.2] for the analogous proof for Wigner matrices)

3.14 $$\begin{aligned} \partial _{\varvec{\alpha }}^{(i)}G_{ba}=-2s_i^3G_{aa}^2G_{bb}^2+{\mathcal {O}}\left( \frac{N^\xi \rho }{N^2\eta }\right) , \end{aligned}$$

with very high probability, where the error comes from terms with at least two off-diagonal *G*’s. Hence we finally conclude that the terms $k=3$ give a contribution:

3.15 $$\begin{aligned} -2\kappa _4\frac{3}{3!}\Vert s\Vert _4^4\frac{1}{N^3}\sum _{ab}G_{aa}^2G_{bb}^2=\frac{\kappa _4}{N}\Vert s\Vert _4^4 m^4+{\mathcal {O}}\left( \frac{N^\xi \rho ^{3/2}}{N^{3/2}\eta ^{1/2}}+\frac{N^\xi \rho }{N^2\eta }\right) . \end{aligned}$$

All the terms with $k\ge 4$ can be estimated trivially using that $|G_{ab}|\lesssim 1$ with very high probability by (3.2). $\square$

### Computation of the variance

For the second moment, using (4.3), we compute

3.16 $$\begin{aligned}{} & {} {\textbf{E}}_H\langle {G_1-{\textbf{E}}_H G_1} \rangle \langle {G_2-{\textbf{E}}_H G_2} \rangle \nonumber \\{} & {} \quad =-{\textbf{E}}_H\left( \frac{m_1'}{m_1}\langle {\underline{s_1^1H_1 G_1}+s_2^1\underline{H_2G_1}} \rangle +\frac{\kappa _4}{N}\Vert s^1\Vert _4^4 m_1'm_1^3\right) \langle {G_2-{\textbf{E}}_H G_2} \rangle +{\mathcal {O}}\left( \frac{N^\xi \Psi _2}{L^{1/2}}\right) \end{aligned}$$

where $s^i=(s_1^i,s_2^i)\in S^1$ and we used (3.10) to approximate $\langle {G_i-{\textbf{E}}_H G_i} \rangle$ with $\langle {G_i-m_i} \rangle$. We made this replacement to use the equation for $G-m$ from (4.3).

Then performing cumulant expansion we compute:

3.17 $$\begin{aligned} \begin{aligned}&-{\textbf{E}}_H\left( \frac{m_1'}{m_1}\langle {s_1^1\underline{H_1 G_1}+s_2^1\underline{H_2G_1}} \rangle +\frac{\kappa _4}{N}\Vert s^1\Vert _4^4 m_1'm_1^3\right) \langle {G_2-{\textbf{E}}_H G_2} \rangle \\&\qquad =\frac{\langle {s^1,s^2} \rangle m_1'{\textbf{E}}_H\langle {G_1G_2^2} \rangle }{m_1N^2}-\frac{\kappa _4}{N}\Vert s^1\Vert _4^4 m_1'm_1^3{\textbf{E}}_H\langle {G_2-{\textbf{E}}_H G_2} \rangle \\&\qquad \quad -\frac{m_1'}{m_1}\sum _{k\ge 2}\sum _{ab}\sum _{{\varvec{\alpha }}\in \{ab,ba\}^k}\left( \frac{\kappa ^{(1)}(ab,{\varvec{\alpha }})}{k!N}s_1^1\partial _{{\varvec{\alpha }}}^{(1)}+s_2^1\frac{\kappa ^{(2)}(ab,{\varvec{\alpha }})}{k!N}\partial _{{\varvec{\alpha }}}^{(2)}\right) \\&\quad {\textbf{E}}_H\big [(G_1)_{ba}\langle {G_2-{\textbf{E}}_H G_2} \rangle \big ]. \end{aligned} \end{aligned}$$

Using the local law (113.11) we conclude that

3.18 $$\begin{aligned} \begin{aligned} \frac{m_1'}{m_1}\langle {s^1,s^2} \rangle \frac{\langle {G_1G_2^2} \rangle }{N^2}&=\langle {s^1,s^2} \rangle \frac{m_1'm_2'}{(1-\langle {s^1,s^2} \rangle m_1m_2)^2N^2}+{\mathcal {O}}\left( \frac{N^\xi }{N^3\eta _1\eta _2\eta _*^2}+\frac{N^\xi }{N^4|\eta _1\eta _2|^3}\right) \\&=-\frac{1}{N^2}\partial _{z_1}\partial _{z_2}\log (1-\langle {s^1,s^2} \rangle m_1m_2) +{\mathcal {O}}\left( \frac{N^\xi }{N^3\eta _1\eta _2\eta _*^2}+\frac{N^\xi }{N^4|\eta _1\eta _2|^3}\right) , \end{aligned} \end{aligned}$$

with very high probability.

We are now left with the third line of (4.11). The ${\varvec{\alpha }}$-derivative in (4.11) may hit either $(G_1)_{ba}$ or $\langle {G_2-{\textbf{E}}_2 G_2} \rangle$. Define

3.19 $$\begin{aligned} \begin{aligned} \Phi _k:&= \frac{m_1'}{m_1}\sum _{ab}\sum _{{\varvec{\alpha }}\in \{ab,ba\}^k} \left( \frac{\kappa ^{(1)}(ab,{\varvec{\alpha }})}{k!N}s_1^1\partial _{{\varvec{\alpha }}}^{(1)} +s_2^1\frac{\kappa ^{(2)}(ab,{\varvec{\alpha }})}{k!N}\partial _{{\varvec{\alpha }}}^{(2)}\right) {\textbf{E}}_H\big [(G_1)_{ba}\langle {G_2-{\textbf{E}}_H G_2} \rangle \big ]\\&=\sum _{ab}\sum _{\varvec{\alpha }}\frac{s_1^1\kappa ^{(1)}(ab,{\varvec{\alpha }})}{k! N}{\textbf{E}}_H\left( \frac{m_1'}{m_1}\partial _{\varvec{\alpha }_1}^{(1)}\frac{(G_1)_{ba}}{k_1!} \right) \left( \partial _{\varvec{\alpha }_2}^{(1)}\frac{\langle {G_2-{\textbf{E}}_H G_2} \rangle }{(k-k_1)!}\right) \\&\quad +\sum _{ab}\sum _{\varvec{\alpha }}\frac{s_2^1\kappa ^{(2)}(ab,{\varvec{\alpha }})}{k! N}{\textbf{E}}_H\left( \frac{m_1'}{m_1}\partial _{\varvec{\alpha }_1}^{(2)}\frac{(G_1)_{ba}}{k_1!}\right) \left( \partial _{\varvec{\alpha }_2}^{(2)}\frac{\langle {G_2-{\textbf{E}}_H G_2} \rangle }{(k-k_1)!}\right) , \end{aligned} \end{aligned}$$

where $k_1$ denotes the number of derivatives that hit $(G_1)_{ba}$. The summation $\sum _{\varvec{\alpha }}$ indicates the summation over tuples ${\varvec{\alpha }}_i^{k_i}$, with $i=1,2$ and $k_2:=k-k_1$. We now claim that

3.20 $$\begin{aligned} \Phi _k=-\varvec{1}(k=3)\Bigl (\kappa _4\frac{\langle {s^1\odot s^1,s^2\odot s^2} \rangle }{2N^2}(m_1^2)'(m_2^2)'+\frac{\kappa _4}{N}\Vert s^1\Vert _4^4 m_1'm_1^3\Bigr )+{\mathcal {O}}\left( N^\xi \frac{\Psi _2}{L^{1/2}}\right) . \end{aligned}$$

Similarly to the proof of [13, Eq. (113)] we readily conclude that the terms in $\Phi _k$ in (4.13) with $k=2$, or $k_1$ odd and $k\ge 4$, or $k\ge 3$ and $k_1$ even are bounded by $N^\xi \Psi _2 L^{-1/2}$. For $k=3$ and $k_1=3$, analogously to (4.8)–(4.9) we obtain a contribution of

3.21 $$\begin{aligned} -\frac{\kappa _4}{N}\Vert s^1\Vert _4^4 m_1'm_1^3+{\mathcal {O}}\left( \frac{N^\xi }{N|\eta _1|L^{1/2}}\right) \end{aligned}$$

to (4.14).

For $k=3$ and $k_1=1$ we start computing the action of the ${\varvec{\alpha }_1}$-derivative on $(G_1)_{ba}$:

3.22 $$\begin{aligned} \sum _{\varvec{\alpha }_1}\partial _{\varvec{\alpha }_1}^{(i)}(G_1)_{ba}=-s_i^1(G_1)_{ba}^2-s_i^1(G_1)_{aa}(G_1)_{bb} =-s_i^1m_1^2(1+\delta _{ab})+{\mathcal {O}}\left( N^\xi \sqrt{\frac{\rho _1}{N|\eta _1|}}\right) , \end{aligned}$$

with very high probability. Additionally, we have that (see [13, Lemma 4.2] for the analogous proof for Wigner matrices)

3.23 $$\begin{aligned} \partial _{ab,ba}^{(i)}\langle {G_2-{\textbf{E}}_H G_2} \rangle =\frac{2 m_2m_2'}{N}(s_i^2)^2 +{\mathcal {O}}\left( \frac{N^\xi \rho _2^{1/2}}{(N|\eta _2|)^{3/2}}\right) , \end{aligned}$$

with very high probability. We thus conclude that the $(k,k_1)=(3,1)$ contribution to (4.14) is

3.24 $$\begin{aligned} -\kappa _4\frac{\langle {s^1\odot s^1,s^2\odot s^2} \rangle }{2N^2}(m_1^2)'(m_2^2)'+{\mathcal {O}}\left( \frac{N^\xi \Psi _2}{L^{1/2}}\right) , \end{aligned}$$

where we used that only the terms with $\kappa _4=\kappa ^{(i)}(ab,ba,ab,ba)$ contribute. This concludes the proof of (3.8) for $p=2$.

### Asymptotic Wick Theorem

The proof of the Wick Theorem for resolvent is completely analogous to the one for Wigner matrices in [13, Section 4]. The only differences are that along the proof we have to carefully keep track of the $s_i$, as we did in Sect. 4.2, since in the Wigner case $s^1=\dots =s^p=(1,0)$, and that we have to use the three *G*’s local law in (3.11) with a weaker error term instead of the one in [13, Eq. (45)] to compute the leading order deterministic term (see (4.21)–(4.22) below).

Define

4.1 $$\begin{aligned} Y_{S}:=\prod _{i\in S} \langle {G_i-{\textbf{E}}_H G_i} \rangle , \end{aligned}$$

with $S\subset {\textbf{N}}$. Similarly to Sect. 4.2 we start computing

4.2 $$\begin{aligned} \begin{aligned} {\textbf{E}}_H Y_{[p]}&=\sum _{i\in [2,p]}\frac{m_1'}{m_1}\frac{\langle {s^1,s^i} \rangle }{N^2} {\textbf{E}}_H\langle {G_1G_i^2} \rangle Y_{[p]{\setminus }\{1,i\}}-\frac{\kappa _4}{N}\Vert s^1\Vert _4^4 m_1'm_1^3 {\textbf{E}}_H Y_{[2,p]} \\&\quad -\sum _{k\ge 2}\sum _{ab}\sum _{{\varvec{\alpha }}\in \{ab,ba\}^k} \left( \frac{\kappa ^{(1)}(ab,{\varvec{\alpha }})}{k!N}s_1^1\partial _{{\varvec{\alpha }}}^{(1)} +s_2^1\frac{\kappa ^{(2)}(ab,{\varvec{\alpha }})}{k!N}\partial _{{\varvec{\alpha }}}^{(2)}\right) {\textbf{E}}_H \left[ \frac{m_1'}{m_1}(G_1)_{ba}Y_{[2,p]}\right] \\&\quad +{\mathcal {O}}\left( N^\xi \frac{\Psi _p}{L^{1/2}}\right) . \end{aligned}\nonumber \\ \end{aligned}$$

Then proceeding analogously to (4.13)–(4.18) (see also [13, Eqs. (110)-(114)] for the Wigner case) we conclude that

4.3 $$\begin{aligned} \begin{aligned} {\textbf{E}}_H Y_{[p]}&=\sum _{i\in [2,p]}\frac{m_1'}{m_1} \frac{\langle {s^1,s^i} \rangle }{N^2}{\textbf{E}}_H\langle {G_1G_i^2} \rangle Y_{[p]{\setminus }\{1,i\}}\\&\quad -\sum _{i\in [2,p]} \kappa _4 \frac{\langle {s^1\odot s^1,s^i\odot s^i} \rangle }{2N^2}(m_1^2)'(m_i^2)'{\textbf{E}}_H Y_{[1,p]{\setminus }\{1,i\}}+{\mathcal {O}}\left( N^\xi \frac{\Psi _p}{L^{1/2}}\right) . \end{aligned} \end{aligned}$$

In order to compute the leading deterministic term of $\langle {G_1G_i^2} \rangle$ we use the local law (3.11) and get

4.4 $$\begin{aligned} {\textbf{E}}_H Y_{[p]}=\frac{1}{N^2}\sum _{i\in [2,p]}V_{1,i}{\textbf{E}}_H Y_{[p]{\setminus }\{1,i\}}+{\mathcal {O}}\left( N^\xi \Psi _p \left( \frac{1}{L^{1/2}}+\frac{1}{N\eta _*^2}+\frac{1}{N^2\eta _*^4}\right) \right) . \end{aligned}$$

Finally, proceeding iteratively we conclude (3.8).

### Multi resolvents local laws

The goal of this section is to prove the local laws in (3.11). Starting from (4.3) we get

4.5 $$\begin{aligned} \begin{aligned} (1-\langle {s^1,s^2} \rangle m_1m_2\langle {\cdot } \rangle )G_1G_2&=m_1m_2+m_1 \langle {G_2-m_2} \rangle -m_1\big (s_1^1\underline{H_1G_1G_2}+s_2^1\underline{H_2G_1G_2}\big ) \\&\quad +m_1\langle {s^1,s^2} \rangle \langle {G_1G_2} \rangle (G_2-m_2)+m_1\langle {G_1-m_1} \rangle G_1G_2. \end{aligned} \end{aligned}$$

We estimate $|\langle {G_1G_2} \rangle |\lesssim N^\xi (\eta ^*)^{-1}$ with very high probability, where $\eta ^*:=\eta _1\vee \eta _2$, using $|\langle {G_1G_2} \rangle | \le \langle {|G_2|} \rangle /\eta _1$ (in case $\eta ^*=\eta _1$) and the rigidity of eigenvalues to estimate $\langle {|G_2|} \rangle \le N^\xi$. Then by the single resolvent local law $|\langle {G_i-m_i} \rangle |\lesssim N^\xi (N\eta _i)^{-1}$ from (3.2) we obtain that

4.6 $$\begin{aligned} (1-\langle {s^1,s^2} \rangle m_1m_2)\langle {G_1G_2} \rangle =m_1m_2-m_1 \big (s_1^1\langle {\underline{H_1G_1G_2}} \rangle +s_2^1\langle {\underline{H_2G_1G_2}} \rangle \big ) +{\mathcal {O}}\left( \frac{N^\xi }{N|\eta _1||\eta _2|}\right) ,\nonumber \\ \end{aligned}$$

with very high probability. Finally, using that

4.7 $$\begin{aligned} |\langle {\underline{H_iG_1G_2}} \rangle |\lesssim \frac{N^\xi }{\sqrt{N|\eta _1\eta _2|}\eta _*}, \qquad i\in [2] \end{aligned}$$

with very high probability from an analogous proof to [15, Eq. (5.8)] (see also [12, Eq. (5.10c)]), and that

4.8 $$\begin{aligned} |1-\langle {s^1,s^2} \rangle m_1m_2|\gtrsim \eta ^*. \end{aligned}$$

we conclude the first local law in (3.11).

For the second local law in (3.11) we start writing the equation for $G_1G_2^2$:

4.9 $$\begin{aligned} \begin{aligned} G_1G_2^2&=m_1m_2'+m_1(G_2^2-m_2')-m_1\big (s_1^1\underline{H_1G_1G_2^2}+s_2^1\underline{H_2G_1G_2^2}\big ) \\&\quad +m_1 \langle {s^1,s^2} \rangle \big (\langle {G_1G_2} \rangle G_2^2+\langle {G_1G_2^2} \rangle G_2\big )+m_1\langle {G_1-m_1} \rangle G_1G_2^2. \end{aligned} \end{aligned}$$

Then, using the usual single *G* local law and the two *G*’s local law from (3.11), we conclude that

4.10 $$\begin{aligned} \begin{aligned} (1-\langle {s^1,s^2} \rangle m_1m_2)\langle {G_1G_2^2} \rangle&=m_1m_2'+ \langle {s^1,s^2} \rangle \frac{m_1^2m_2m_2'}{1 -\langle {s^1,s^2} \rangle m_1m_2} \\&\quad -m_1\big (s_1^1\underline{H_1G_1G_2^2}+s_2^1\underline{H_2G_1G_2^2}\big ) +{\mathcal {O}}\left( \frac{N^\xi }{N|\eta _1||\eta _2|\eta _*}\right) . \end{aligned} \end{aligned}$$

Then, using that

4.11 $$\begin{aligned} |\langle {\underline{H_iG_1G_2^2}} \rangle |\lesssim \frac{N^\xi }{N\sqrt{|\eta _1\eta _2|}\eta _*^2}, \qquad i\in [2], \end{aligned}$$

with very high probability, and (4.26) we conclude (3.11). The proof of (4.29) follows analogously to the one of (4.25).

## Stationary Phase Calculations

The proof of (2.17) is a tedious stationary phase calculation since $v_\pm ^{sr}(t)$, the leading part of $v_{\pm ,\kappa }^{sr}(t)$ (see (2.12)), are given in terms of oscillatory integrals for $t\gg 1$ being the large parameter. Unlike in the $s=r$ case, no explicit formula similar to (2.21) is available. The main complication is that $V^{sr}(x,y)$ defined in (2.7) has logarithmic singularities, integrated against a fast oscillatory term from $f'g'$, so standard stationary phase formulas cannot directly be applied. Nevertheless, a certain number of integration by parts can still be performed before the derivative of the integrand stops being integrable and the leading term can be computed.

We will first give a proof of

4.12 $$\begin{aligned} {\textbf{E}}_s{\textbf{E}}_r v^{sr}_-(t) \sim \sqrt{t} \end{aligned}$$

then we explain how to modify this argument to obtain

4.13 $$\begin{aligned} {\textbf{E}}_s{\textbf{E}}_r v^{sr}_-(t)^2 \sim t^{3/2}, \end{aligned}$$

in both cases with a definite large *t* asymptotics with computable explicit constants. The proof reveals that the corresponding results for ${\textbf{E}}_s{\textbf{E}}_r v^{sr}_+(t)$ and ${\textbf{E}}_s{\textbf{E}}_r v^{sr}_+(t)^2$ guarantee only an upper bound with the same behavior

4.14 $$\begin{aligned} {\textbf{E}}_s{\textbf{E}}_r v^{sr}_+(t) \lesssim \sqrt{t}, \qquad {\textbf{E}}_s{\textbf{E}}_r v^{sr}_+(t)^2 \lesssim t^{3/2} \end{aligned}$$

depending on the distribution of *s* on $S^1$, the matching lower bound may not necessarily hold. However, for our main conclusions like (2.19) only an upper bound on $S_\textrm{res}(t)$ is important.

All these exponents are valid for the $k=2$ case, i.e. for $H^s=s_1H_1+s_2H_2$. For the general multivariate model, $k\ge 3$, exactly the same proof gives the upper bounds

4.15 $$\begin{aligned} {\textbf{E}}_s{\textbf{E}}_r v^{sr}_\pm (t) \lesssim \min \{ 1, t^{\frac{3-k}{2}} \}, \qquad {\textbf{E}}_s{\textbf{E}}_r v^{sr}_+(t)^2 \lesssim \min \{ 1, t^{\frac{5-k}{2}} \}. \end{aligned}$$

The *k*-dependence of the exponent can directly be related to the tail behavior (5.5) and (5.8) below, so for simplicity we will carry out our main analysis only for $k=2$. In fact, a more careful analysis yields somewhat better bounds than (5.4), but we will not pursue this improvement here.

We introduce a new random variable

$$\begin{aligned} U: = \langle s, r\rangle \end{aligned}$$

then clearly $|U|\le 1$ and since $r, s\in S^k$ have a distribution with an $L^2$ density, it is easy to see that the density $\rho ^*$ of *U* is bounded by

4.16 $$\begin{aligned} \rho ^* (U)\lesssim (1-U^2)^{\frac{k-3}{2}}. \end{aligned}$$

The fact that the main contribution to the lhs. of (5.4) comes from the regime $U\approx 1$ is a consequence of the singularity of the logarithm in (2.7) in this regime (see computations below). Indeed, $U=\cos \alpha$ where $\alpha$ is the angle between *r*, *s* and near $U\approx \pm 1$ we have $1\pm U \approx \frac{1}{2}\alpha ^2(1+ O(\alpha ^2))$. For example, for $k=2$ we have

4.17 $$\begin{aligned}{} & {} {\textbf{P}}( 1-U= \epsilon +{\text {d}}\!{}\epsilon ) = \frac{{\text {d}}\!{}\epsilon }{\sqrt{\epsilon }} \Big (\int _{S^1}\rho ^2(s){\text {d}}\!{}s\Big )(1+O(\sqrt{\epsilon })) \end{aligned}$$

4.18 $$\begin{aligned}{} & {} {\textbf{P}}( 1+U= \epsilon +{\text {d}}\!{}\epsilon ) = \frac{{\text {d}}\!{}\epsilon }{\sqrt{\epsilon }} \Big (\int _{S^1}\rho (s)\rho (s+\pi ){\text {d}}\!{}s \Big )(1+O(\sqrt{\epsilon })) \end{aligned}$$

in the $\epsilon \ll 1$ regime. In particular, the bound in (5.5) is actually an asymptotics in the most critical $U\approx 1$ regime, while the regime $U\approx -1$ it may happen that the density $\rho ^*$ is much smaller than (5.5) predicts. For symmetric distribution, $\rho (s)=\rho (s+\pi )$, the two asymptotics are the same. Similar relations hold for $k\ge 3$, in which case we have

4.19 $$\begin{aligned} {\textbf{P}}( 1\pm U = \epsilon +{\text {d}}\!{}\epsilon ) \lesssim \epsilon ^{\frac{k-3}{2}}{\text {d}}\!{}\epsilon \end{aligned}$$

with an explicit asymptotics for $U\approx 1$.

So we will study

4.20 $$\begin{aligned} R_\pm (t) = t^2 \Re \int {\text {d}}\!{}U \rho ^*(U) \iint _{-2}^2 {\text {d}}\!{}x{\text {d}}\!{}y e^{\textrm{i}t (x\pm y)} \Big [ \log |{1-U m(x)\overline{m(y})} | - \log |{1-Um(x)m(y)} |\Big ]. \end{aligned}$$

Since $|m|\le 1$, as long as $|U|\le 1-\delta$ for any small fixed $\delta >0$, the arguments of the logarithms are separated away from zero and they allow to perform arbitrary number of integration by parts, each gaining a factor of 1/*t*. There is a square root singularity of *m*(*x*) and *m*(*y*) at the spectral edges $2, -2$ which still allows one to perform one integration by parts in each variable since $m'$ is still integrable. Therefore the contribution of the regime $|U|\le 1-\delta$ to (5.9) is of order $t^2(1/t)^2= O(1)$, hence negligible compared with the target (5.1). In the sequel we thus focus on the important $U\approx \pm 1$ regimes, in particular every $\int {\text {d}}\!{}U$ integral is understood to be restricted to $|U|\ge 1-\delta$.

Note that $\overline{m(y)} =-m(-y)$, so if *U* has a symmetric distribution (for example if $s\in S^1$ has a symmetric distribution), then by symmetry we have

$$\begin{aligned} R_-(t)=-R_+(t). \end{aligned}$$

For definiteness, we focus on $R_-(t)$, the analysis of $R_+$ is analogous. From the explicit form $m(x) =\frac{1}{2}(-x+\textrm{i}\sqrt{4-x^2})$ a simple exercise shows that

4.21 $$\begin{aligned} |1-Um(x)\overline{m(y)}|^2 \gtrsim (1-U)^2 + (x-y)^2, \qquad |1-Um(x) m(y)|^2 \gtrsim (1+U)^2 + (x+y)^2. \end{aligned}$$

This shows that the critical regime is $U\approx 1$ and $x\approx y$ for the first integrand in (5.9) and $U\approx -1$, $x\approx -y$ for the second. Again, for definiteness, we focus on the first regime, i.e. on the first log-integrand in (5.9) and establish the following relations for large *t* and $k=2$:

### Lemma 5.1

In the $k=2$ case we have

4.22 $$\begin{aligned} t^2 \int {\text {d}}\!{}U \rho ^*(U) \Re \iint _{-2}^2 e^{\textrm{i}t (x- y)} \log |{1-U m(x)\overline{m(y})} |^2 {\text {d}}\!{}x {\text {d}}\!{}y \sim \sqrt{t} \end{aligned}$$

and

4.23 $$\begin{aligned} t^4 \int {\text {d}}\!{}U \rho ^*(U) \Bigg [ \Re \iint _{-2}^2 e^{\textrm{i}t (x- y)} \log |{1-U m(x)\overline{m(y})} |^2 {\text {d}}\!{}x {\text {d}}\!{}y \Bigg ]^2 \sim t^{3/2}, \end{aligned}$$

for $t\ge 1$. For $t\gg 1$ an analogous asymptotic statement holds with explicitly computable positive constants that depend on the distribution of *s*.

### Proof of Lemma 5.1

Introduce the variables

$$\begin{aligned} a:=\frac{x+y}{2}, \quad b:=\frac{x-y}{2}, \qquad \text{ i.e. } \quad x= a+b, \quad y= a-b. \end{aligned}$$

Since $|x|, |y|\le 2$ we have

4.24 $$\begin{aligned} |a|\le 2, \qquad |b|\le \min \{ |2-a|, |2+a|\}. \end{aligned}$$

In terms of these variables, we have

4.25 $$\begin{aligned}{} & {} |1-Um(x)\overline{m(y)}|^2 = \Big ( 1- U + 2U \frac{b^2}{ b^2 + d^2} \Big )^2 + \frac{4 U^2 b^2 d^2}{(b^2+ d^2)^2}, \qquad \nonumber \\{} & {} \quad d:= \frac{1}{2}\big [ \sqrt{4-(a+b)^2} +\sqrt{4-(a-b)^2}\big ]. \end{aligned}$$

Here we also used the identity

$$\begin{aligned} 1-m(x)\overline{m(y)} = \frac{2b}{2b + m(x)-\overline{m(y)}} =\frac{2b}{b+\textrm{i}d} \end{aligned}$$

following from the equation $-m(x)^{-1}=x+ m(x)$ and similarly for *m*(*y*). In the regime (5.13) we have

4.26 $$\begin{aligned} |b|\le \frac{1}{2}(4-a^2), \qquad |b|\le \sqrt{4-a^2}. \end{aligned}$$

Note that by Taylor expansion around *a* and concavity of the function $x\rightarrow \sqrt{4-x^2}$ in $x\in [-2,2]$, we have

4.27 $$\begin{aligned} 0\le \sqrt{4-a^2}-d \lesssim \frac{b^2}{(4-a^2)^{3/2}}\le \frac{|b|}{\sqrt{4-a^2}}, \quad \text{ as } \text{ well } \text{ as } \quad \frac{1}{2}\sqrt{4-a^2}\le d\le \sqrt{4-a^2}. \end{aligned}$$

We define the function

4.28 $$\begin{aligned} F = F(U, a, b): = (1-U)^2 +\frac{4U^2b^2}{4-a^2} \end{aligned}$$

for $|U|\le 1$, and *a*, *b* as in (5.13). We will use *F* to approximate

4.29 $$\begin{aligned} M=M(U, a,b):=|1-Um(a+b)\overline{m(a-b)}|^2 \end{aligned}$$

in the critical regime where $|U|\ge 1-\delta$ and $|b|\le \delta$ for some small fixed $\delta >0$. We clearly have

5.1 $$\begin{aligned} M(U, a,b)\ge \frac{1}{4} F(U, a, b) \end{aligned}$$

in the regime (5.13), where $|b|\le \sqrt{4-a^2} \le 2d$, using (5.16).

For the difference function

5.2 $$\begin{aligned} \Delta (U, a, b):= M(U, a,b)- F(U, a, b) \end{aligned}$$

an elementary calculation from (5.14)–(5.16) gives

5.3 $$\begin{aligned} \big | \Delta (U, a, b)\big | \lesssim \frac{b^2}{(4-a^2)^{3/2}} \sqrt{F} \end{aligned}$$

in the regime $|U|\ge 1-\delta$ and $|b|\le \delta$. Furthermore, similar estimates hold for the first derivative;

5.4 $$\begin{aligned} \Bigg | \frac{{\text {d}}\!{}}{{\text {d}}\!{}b}\Delta (U, a, b)\Big ]\Bigg | \lesssim \frac{|b|\sqrt{F}}{(4-a^2)^{3/2}}, \quad \Bigg | \frac{{\text {d}}\!{}}{{\text {d}}\!{}a}\Delta (U, a, b)\Big ]\Bigg | \lesssim \frac{b^2\sqrt{F}}{(4-a^2)^{5/2}} \lesssim \frac{|b|\sqrt{F}}{(4-a^2)^{3/2}}, \end{aligned}$$

as well as for the second derivatives

5.5 $$\begin{aligned} \Bigg | \frac{{\text {d}}\!{}^2 }{{\text {d}}\!{}b^2}\Delta (U, a, b)\Big ]\Bigg | \lesssim \frac{\sqrt{F}}{(4-a^2)^{3/2}}, \quad \Bigg | \frac{{\text {d}}\!{}}{{\text {d}}\!{}a}\frac{{\text {d}}\!{}}{{\text {d}}\!{}b}\Delta (U, a, b)\Big ]\Bigg | \lesssim \frac{|b|\sqrt{F}}{(4-a^2)^{5/2}} \lesssim \frac{\sqrt{F}}{(4-a^2)^{3/2}}. \end{aligned}$$

The proof of Lemma 5.1 consists of two parts. First we compute the integral with $\log F$, i.e. we show that

5.6 $$\begin{aligned} t^2 \int {\text {d}}\!{}U \rho ^*(U) \Re \iint _{-2}^2 e^{\textrm{i}t (x- y)} \log F\big (U, \frac{x+y}{2}, \frac{x-y}{2}\big ){\text {d}}\!{}x {\text {d}}\!{}y \sim \sqrt{t} \end{aligned}$$

with an explicit positive constant factor in the asymptotic regime $t\gg 1$. Second, we show that the integrand in (5.11) can indeed be replaced with *F* up to a negligible error,

5.7 $$\begin{aligned} \Bigg | t^2 \int {\text {d}}\!{}U \rho ^*(U) \iint _{-2}^2 e^{\textrm{i}t (x- y)} \Big [ \log |{1-U m(x)\overline{m(y})} |^2 - \log F\big (U, \frac{x+y}{2}, \frac{x-y}{2}\big )\Big ] {\text {d}}\!{}x {\text {d}}\!{}y\Bigg | \lesssim 1. \end{aligned}$$

**Part I.** To prove (5.24), we use the *a*, *b* variables and the symmetry of *F* in *a* to restrict the *a* integration to $0\le a\le 2$:

5.8 $$\begin{aligned} (5.24) =4t^2 \Re \int {\text {d}}\!{}U \rho ^*(U) \int _0^2 {\text {d}}\!{}a \int _{-(2-a)}^{2-a} {\text {d}}\!{}b\; e^{2\textrm{i}t b} \log F\big (U,a,b). \end{aligned}$$

Using integration by parts, we have

5.9 $$\begin{aligned} \begin{aligned} \int _{-(2-a)}^{2-a} {\text {d}}\!{}b\; e^{2\textrm{i}t b} \log \big [ (1-U)^2 +\frac{4U^2b^2}{4-a^2}\big ]&= \frac{1}{2\textrm{i}t} \Big [ e^{2\textrm{i}t (2-a)} - e^{-2\textrm{i}t (2-a)}\Big ] \log \big [ (1-U)^2 +\frac{4U^2(2-a)}{2+a}\big ] \\&\quad - \frac{1}{2\textrm{i}t} \frac{4U^2}{4-a^2} \int _{-(2-a)}^{2-a} {\text {d}}\!{}b\; e^{2\textrm{i}t b} \frac{ 2b}{ (1-U)^2 +\frac{4U^2b^2}{4-a^2} }. \end{aligned} \end{aligned}$$

In the boundary terms we can perform one more integration by parts in the *a* variable when plugged into (5.26). Just focusing on the first boundary term in (5.27), using $|U|\le 1$ we have

$$\begin{aligned}{} & {} \Bigg | \frac{1}{2\textrm{i}t} e^{4\textrm{i}t} \int _0^2 {\text {d}}\!{}a \, e^{-2\textrm{i}t a}\log \big [ (1-U)^2 +\frac{4U^2(2-a)}{2+a}\big ] \Bigg | \\{} & {} \quad \lesssim \frac{1}{t^2} \int _0^2 \frac{{\text {d}}\!{}a}{ (1-U)^2 + U^2(2-a)} \lesssim \frac{|\log (1-U)|}{t^2}. \end{aligned}$$

Since $\rho ^*(U)$ is a density bounded by $(1-U^2)^{-1/2}$ in the $U\approx 1$ regime from (5.5), the logarithmic singularity is integrable showing that the two boundary terms in (5.27), when plugged into (5.26), give at most an *O*(1) contribution, negligible compared with the target behavior of order $\sqrt{t}$ in (5.1).

To compute the main (second) term in the rhs. of (5.27), we first extend the integration limits to infinity and claim that

5.10 $$\begin{aligned} \begin{aligned}&t^2 \int {\text {d}}\!{}U \rho ^*(U) \Big | \frac{1}{2\textrm{i}t} \int _0^2{\text {d}}\!{}a \frac{4U^2}{4-a^2} \int _{2-a}^{\infty } {\text {d}}\!{}b\; e^{2\textrm{i}t b} \frac{ 2b}{ (1-U)^2 +\frac{4U^2b^2}{4-a^2} }\Big |\\&\quad \lesssim t \int {\text {d}}\!{}U \rho ^*(U)\int _0^2\frac{{\text {d}}\!{}a}{2-a}\Bigg | \int _{2-a}^{\infty } {\text {d}}\!{}b\; e^{2\textrm{i}t b} \frac{ 2b}{ (1-U)^2 +\frac{4U^2b^2}{4-a^2} }\Bigg | \end{aligned} \end{aligned}$$

gives a negligible contribution to (5.26) (the lower limit is removed similarly). Indeed, we apply one more integration by parts inside the absolute value in (5.28):

$$\begin{aligned} \Bigg | \int _{2-a}^{\infty } {\text {d}}\!{}b\; e^{2\textrm{i}t b} \frac{ 2b}{ (1-U)^2 +\frac{4U^2b^2}{4-a^2} }\Bigg | \lesssim t^{-1} \int _{2-a}^{\infty } \frac{{\text {d}}\!{}b}{ (1-U)^2 +\frac{U^2b^2}{4-a^2} } + t^{-1} \frac{ 2-a }{ (1-U)^2 +(2-a) }. \end{aligned}$$

Its contribution to the rhs of (5.28) is thus bounded by

$$\begin{aligned}{} & {} \int {\text {d}}\!{}U \rho ^*(U) \int _0^2\frac{{\text {d}}\!{}a}{2-a} \Big [ \int _{2-a}^{\infty } \frac{{\text {d}}\!{}b}{ (1-U)^2 +\frac{U^2b^2}{4-a^2} } + \frac{ 2-a }{ (1-U)^2 +(2-a) }\Big ]\\{} & {} \quad \lesssim \int \frac{{\text {d}}\!{}U}{\sqrt{1-U^2}}\Bigg [ \int _0^2 \frac{{\text {d}}\!{}a}{\sqrt{2-a} }\frac{1}{1-U + \sqrt{2-a}} + |\log (1-U)|\Bigg ] \lesssim 1. \end{aligned}$$

Summarizing, we just proved that

11 $$\begin{aligned} \begin{aligned} (5.24)&= -2t \Im \int {\text {d}}\!{}U \rho ^*(U) \int _0^2 {\text {d}}\!{}a \frac{4U^2}{4-a^2} \int _{-\infty }^\infty {\text {d}}\!{}b\; e^{2\textrm{i}t b} \frac{ 2b}{ (1-U)^2 +\frac{4U^2b^2}{4-a^2} } +O(1)\\&= \frac{t}{\pi } \int {\text {d}}\!{}U \rho ^*(U) \int _0^2 {\text {d}}\!{}a \; e^{-t\sqrt{4-a^2}(1-U)/U} +O(1)\\&= \frac{c_0t}{\pi } \int {\text {d}}\!{}U \frac{1}{\sqrt{1-U}} \int _0^2 {\text {d}}\!{}a \; e^{-t\sqrt{4-a^2}(1-U)/U} +O(1)\\&= \frac{c_0\sqrt{t}}{\pi } \int _0^\infty \frac{e^{-v}}{\sqrt{v}} {\text {d}}\!{}v \int _0^2 \frac{{\text {d}}\!{}a}{(4-a^2)^{1/4}} +O(1) \\&= \frac{\Gamma (3/4)}{\sqrt{2}\Gamma (5/4)}c_0 \sqrt{t} + O(1), \end{aligned} \end{aligned}$$

where in the second line we used residue calculation, in the third line we used that

$$\begin{aligned} \rho ^*(U) = \frac{c_0}{\sqrt{1-U}} +O(1) \end{aligned}$$

in the regime $U\approx 1$ with some positive constant $c_0>0$ depending on the distribution of *s* (see (5.6)), and finally in the fourth line we used that for large *t* the main contribution to the integral comes from $U\approx 1$ in order to simplify the integrand. This completes the proof of (5.24).

**Part II.** We now prove (5.25). After changing to the *a*, *b* variables and considering only the $0\le a\le 2$ regime for definiteness, we perform an integration by parts in *b* that gives

5.11 $$\begin{aligned} \begin{aligned} (5.25) \lesssim&\; t \int {\text {d}}\!{}U \rho ^*(U) \Bigg | \int _{0}^2 {\text {d}}\!{}a \, e^{2\textrm{i}ta} \Big [ \log M(U, a,b) -\log F\big (U, a, b\big ) \Big ] {\text {d}}\!{}b\Bigg | \\&+ t \int {\text {d}}\!{}U \rho ^*(U) \int _{0}^2 {\text {d}}\!{}a \Bigg | \int _{-(2-a)}^{2-a} e^{2\textrm{i}tb} \partial _b\Big [ \log M(U, a,b) -\log F\big (U, a, b\big ) \Big ] {\text {d}}\!{}b\Bigg | \end{aligned} \end{aligned}$$

recalling the definition of *M* from (5.18). The first term in (5.30) is the boundary term, which is negligible after one more integration by parts using the $\partial _a$ derivative estimate from (5.22).

In the second term we perform one more integration by parts to obtain

12 $$\begin{aligned} \begin{aligned} (5.25) \lesssim&\; t \int {\text {d}}\!{}U \rho ^*(U) \Bigg | \int _{0}^2 {\text {d}}\!{}a \, e^{2\textrm{i}ta} \partial _b\Big [ \log M(U, a,b) -\log F\big (U, a, b\big ) \Big ] {\text {d}}\!{}b\Bigg | \\&+\int {\text {d}}\!{}U \rho ^*(U) \int _{0}^2 {\text {d}}\!{}a \int _{-(2-a)}^{2-a}\Bigg | \partial _b^2\Big [ \log M \big (U, a, b\big )-\log F\big (U, a, b\big ) \Big ] \Bigg | {\text {d}}\!{}b, \end{aligned} \end{aligned}$$

where the first term comes from the boundary. In this term we can perform one more integration by parts in *a*. The corresponding boundary terms are easily seen to be order one and the main term is analogous to the first term in the rhs of (5.31) just we have the mixed $\partial _a\partial _b$ derivative. Recalling $\Delta = M-F$ from (5.20), we use the estimate

$$\begin{aligned} \big | \partial _b^2 [\log M- \log F]\big | \lesssim \frac{|\partial _b^2 \Delta | }{F} + \frac{|\partial _b^2 F|}{F^2} |\Delta |+ \frac{|\partial _b \Delta ||\partial _b M +\partial _b F| }{F^2} + (\partial _bF)^2\frac{|\Delta |}{F^3} \end{aligned}$$

in the situation where $M\gtrsim F>0$ are positive functions (see (5.19)). Similar bound holds for the mixed derivative.

Therefore, we can estimate both integrals in (5.31) as follows:

5.12 $$\begin{aligned} \begin{aligned} (5.25) \lesssim&\int {\text {d}}\!{}U \rho ^*(U) \int _{0}^2 {\text {d}}\!{}a \int _{-(2-a)}^{2-a} \frac{1}{(4-a^2)^{3/2}} \frac{1}{\big [ (1-U)^2 + \frac{b^2}{4-a^2}\big ]^{1/2}} {\text {d}}\!{}b \\ \lesssim&\int \frac{{\text {d}}\!{}U}{\sqrt{1-U^2}} \int _{0}^2 \frac{{\text {d}}\!{}a}{4-a^2} \int _0^{\sqrt{2-a}} \frac{{\text {d}}\!{}u}{ \big [ (1-U)^2 + u^2\big ]^{1/2}}\\ \lesssim&\int \frac{{\text {d}}\!{}U}{\sqrt{1-U^2}} \int _0^{\sqrt{2}} \frac{ |\log u|+1 }{ \big [ (1-U)^2 + u^2\big ]^{1/2}} {\text {d}}\!{}u\\ \lesssim&\int \frac{{\text {d}}\!{}U |\log (1-U)|^2}{(1-U)^{1/2}} \lesssim 1. \end{aligned} \end{aligned}$$

Here we used the bounds (5.21), (5.22) and (5.23) and that $|b|\le 2-a\lesssim 4-a^2$ to simplify some estimates. For computing the derivatives of *F* we used its explicit form (5.17). This completes the proof of (5.25) and thus also the proof of (5.11) in Lemma 5.1.

The proof of (5.12) is very similar. We again approximate $M=|1-Um(x)\overline{m(y)}|^2$ by *F* at the expense of negligible errors. We omit these calculations as they are very similar those for (5.11) and focus only on the main term which is (see the analogous (5.26))

5.13 $$\begin{aligned} 16t^4 \int {\text {d}}\!{}U \rho ^*(U) \Big [ \Re \int _0^2 {\text {d}}\!{}a \int _{-(2-a)}^{2-a} {\text {d}}\!{}b \; e^{2\textrm{i}tb} \log F(U, a, b)\Big ]^2. \end{aligned}$$

After one integration by parts and neglecting the lower order boundary terms, we have the following analogue of (5.29):

5.14 $$\begin{aligned} \begin{aligned} 4t^2 \int {\text {d}}\!{}U \rho ^*(U)&\Big [ \Re \int _0^2 {\text {d}}\!{}a \frac{U^2}{4-a^2} \int _{-\infty }^\infty {\text {d}}\!{}b\; e^{2\textrm{i}t b} \frac{ 2b}{ (1-U)^2 +\frac{4U^2b^2}{4-a^2} } \Big ]^2\\&= \frac{t^2}{\pi ^2} \int {\text {d}}\!{}U \rho ^*(U) \Big [ \int _0^2 {\text {d}}\!{}a \; e^{-t\sqrt{4-a^2}(1-U)/U}\Big ]^2 \\&\approx \frac{c_0 t^{3/2}}{\pi ^2} \int _0^\infty \frac{{\text {d}}\!{}v}{\sqrt{v}} \Big ( \int _0^2 {\text {d}}\!{}a \; e^{-\sqrt{4-a^2}v } \Big )^2 \\&= \frac{c_0 t^{3/2}}{\pi ^2} \iint _0^2 \frac{{\text {d}}\!{}a_1{\text {d}}\!{}a_2}{(\sqrt{4-a_1^2} +\sqrt{4-a_2^2})^{1/2}} \int _0^\infty \frac{e^{-v}}{\sqrt{v}} {\text {d}}\!{}v \sim t^{3/2} \end{aligned} \end{aligned}$$

as the leading term. This proves (5.12) and completes the proof of Lemma 5.1. $\square$

We close this section by commenting on the proof of the upper bound in (2.28). Recall from (2.16) that the essential part of $\widetilde{S}_\textrm{res}(t)$ in the slope regime is given by ${\textbf{E}}_s {\textbf{E}}_r \widetilde{v}^{sr}(t)$ expressed by the oscillatory integrals

5.15 $$\begin{aligned} R_\pm (t):=t^2 \iint _{{\textbf{R}}^2} \rho (s)\rho (r){\text {d}}\!{}s {\text {d}}\!{}r \iint _{-2}^2 {\text {d}}\!{}x{\text {d}}\!{}y e^{\textrm{i}t (\Vert s\Vert x\pm \Vert r\Vert y)} A(U, x, y) \end{aligned}$$

with

$$\begin{aligned} A(U, x, y): = \log |{1-U m(x)\overline{m(y})} | - \log |{1-Um(x)m(y)} |, \end{aligned}$$

where $U= \frac{\langle s, r\rangle }{\Vert s\Vert \Vert r \Vert }$ is the cosine of the angle between the vectors $s,r\in {\textbf{R}}^2$. Assuming for the moment that $\rho$, the density of *s*, is rotationally symmetric, $\rho (s)= \rho (\Vert s\Vert )$ with a slight abuse of notations, we have

5.16 $$\begin{aligned} \begin{aligned} R_\pm (t) \sim&\; t^2 \int _{-1}^1 \frac{{\text {d}}\!{}U}{\sqrt{1-U^2}} \iint _{-2}^2 {\text {d}}\!{}x{\text {d}}\!{}y A(U, x, y) \int _0^\infty e^{\textrm{i}t x \sigma }\rho (\sigma )\sigma {\text {d}}\!{}\sigma \int _0^\infty e^{\pm \textrm{i}t y \sigma '}\rho (\sigma ')\sigma ' {\text {d}}\!{}\sigma ' \\ \sim&\; t \int _{-1}^1 \frac{{\text {d}}\!{}U}{\sqrt{1-U^2}} \iint _{-2}^2 {\text {d}}\!{}x{\text {d}}\!{}y \widehat{\rho }(tx) \widehat{\rho (\sigma )\sigma }(\pm ty) \frac{{\text {d}}\!{}}{{\text {d}}\!{}x} A(U, x, y)\\ \end{aligned} \end{aligned}$$

performing an integration by parts in *x* and ignoring lower order boundary term. In the last step we also computed the Fourier transform (we used that $\rho (0)=0$ to extend $\rho$ to ${\textbf{R}}$). The main contribution comes from the regime where *A* is nearly singular, and considering (5.10), we just focus on the regime $U\sim 1$ and $x\sim y$, the singularity from the other logarithmic term is treated analogously. Similarly to the proof of (5.25) we may ignore the edge regime, and effectively we have

5.17 $$\begin{aligned} \Big | \frac{{\text {d}}\!{}}{{\text {d}}\!{}x} \log |{1-U m(x)\overline{m(y})} | \Big | \lesssim \frac{1}{(1-U)+ |x-y|}. \end{aligned}$$

Thus we can continue estimating the last line of (5.36)

$$\begin{aligned} |(5.36)|\lesssim t \int _{-1}^1 \frac{{\text {d}}\!{}U}{\sqrt{1-U^2}} \iint _{-2}^2 {\text {d}}\!{}x{\text {d}}\!{}y \frac{\big | \widehat{\rho }(tx) \widehat{\rho (\sigma )\sigma }(ty)\big | }{ (1-U)+ |x-y|} \lesssim t^{-1/2}. \end{aligned}$$

Here we used the regularity of $\rho$, so that the last two factors essentially restrict the integration to the regime $|x|, |y|\lesssim 1/t$. The final inequality is obtained just by scaling.

To understand $\widetilde{S}_\textrm{res}(t)$ in the ramp regime, we need to compute ${\textbf{E}}_s {\textbf{E}}_r \widetilde{v}^{sr}_\pm (t)^2$, i.e. integrals of the following type:

5.18 $$\begin{aligned} \begin{aligned} t^4 \iint _{{\textbf{R}}^2}&\rho (s)\rho (r){\text {d}}\!{}s {\text {d}}\!{}r \Bigg | \iint _{-2}^2 {\text {d}}\!{}x{\text {d}}\!{}y e^{\textrm{i}t (\Vert s\Vert x\pm \Vert r\Vert y)} A(U, x, y) \Bigg |^2\\ =&\, t^4 \int _{-1}^1 \frac{{\text {d}}\!{}U}{\sqrt{1-U^2}} \iint _{-2}^2 {\text {d}}\!{}x{\text {d}}\!{}y \iint _{-2}^2 {\text {d}}\!{}x'{\text {d}}\!{}y' A(U, x, y)\overline{A(U, x', y')} \\&\times \int _0^\infty e^{\textrm{i}t (x-x') \sigma }\rho (\sigma )\sigma {\text {d}}\!{}\sigma \int _0^\infty e^{\pm \textrm{i}t (y-y') \sigma '}\rho (\sigma ')\sigma ' {\text {d}}\!{}\sigma '\\ \sim&\, t^2 \int _{-1}^1 \frac{{\text {d}}\!{}U}{\sqrt{1-U^2}} \iint _{-2}^2 {\text {d}}\!{}x{\text {d}}\!{}y \iint _{-2}^2 {\text {d}}\!{}x'{\text {d}}\!{}y' \frac{{\text {d}}\!{}}{{\text {d}}\!{}x} A(U, x, y) \frac{{\text {d}}\!{}}{{\text {d}}\!{}y'} \overline{A(U, x', y')} \\&\times \int _0^\infty e^{\textrm{i}t (x-x') \sigma }\rho (\sigma ){\text {d}}\!{}\sigma \int _0^\infty e^{\pm \textrm{i}t (y-y') \sigma '}\rho (\sigma ') {\text {d}}\!{}\sigma ' \\ \sim&\, t^2 \int _{-1}^1 \frac{{\text {d}}\!{}U}{\sqrt{1-U^2}} \iint \!\!\!\iint _{-2}^2 {\text {d}}\!{}x{\text {d}}\!{}y {\text {d}}\!{}x'{\text {d}}\!{}y' \widehat{\rho }(t(x-x'))\widehat{\rho }(\pm t(y-y')) \frac{{\text {d}}\!{}}{{\text {d}}\!{}x} \\&\quad A(U, x, y) \frac{{\text {d}}\!{}}{{\text {d}}\!{}y'} \overline{A(U, x', y')}. \end{aligned} \end{aligned}$$

Here we performed two integrations by parts in *x* and $y'$ and ignored the boundary terms. Estimating the derivative of *A* as in (5.37), we can continue

$$\begin{aligned}{} & {} |(5.38)|\lesssim t^2 \int _{-1}^1 \frac{{\text {d}}\!{}U}{\sqrt{1-U^2}} \iint _{-2}^2 \frac{{\text {d}}\!{}x{\text {d}}\!{}y }{(1-U)+ |x-y|} \\{} & {} \quad \iint _{-2}^2 \frac{{\text {d}}\!{}x'{\text {d}}\!{}y' }{(1-U)+ |x'-y'|} \big |\widehat{\rho }(t(x-x'))\widehat{\rho }(t(y-y'))\big |. \end{aligned}$$

The last two factors essentially restrict the integration to the regime $|x-x'|\lesssim 1/t$, $|y-y'|\lesssim 1/t$ and by scaling we obtain a bound of order $t^{1/2}$ for |(5.38)|. This completes the sketch of the proof of (2.28) in the radially symmetric case, the general case is analogous but technically more cumbersome and we omit the details.

## Data Availability

All data generated or analysed are included in this published article.

## Footnotes

1 Equation (1.7) is for matrices whose second and fourth moments coincide with the ones of GUE, otherwise there are additional terms, see e.g. [13, Theorem 2.4].

2 We assume equal fourth cumulants merely for notational convenience. Our proof verbatim covers also the more general case.

3 For the applications in this paper, SFF in the regime $t\gg 1$, the first term in (2.7) is the only relevant one.

4 The exponent in (2.8) can be optimized depending on $\tau$ and $f$.

5 The resolvent method extends to very general Hermitian matrices possibly with non-centered and correlated entries, see [19], but here we present only the Wigner case for simplicity.
